# Ultrananocrystalline Diamond Nanowires: Fabrication, Characterization, and Sensor Applications

**DOI:** 10.3390/ma14030661

**Published:** 2021-01-31

**Authors:** Andrew F. Zhou, Xinpeng Wang, Elluz Pacheco, Peter X. Feng

**Affiliations:** 1 Department of Physics, Indiana University of Pennsylvania, Indiana, PA 15705, USA; 2 Department of Physics, University of Puerto Rico, San Juan, PR 00936, USA; david011608@gmail.com (X.W.); elluz.pacheco@upr.edu (E.P.)

**Keywords:** ultrananocrystalline diamond (UNCD), boron doping, nitrogen doping, nanowire (NW), gas sensor, ultraviolet (UV), photodetector (PD), nanoplasmonic, piezoresistance (PZR), biosensor, nitrogen-vacancy (NV), magnetic field quantum sensor

## Abstract

The aim of this review is to provide a survey of the recent advances and the main remaining challenges related to the ultrananocrystalline diamond (UNCD) nanowires and other nanostructures which exhibit excellent capability as the core components for many diverse novel sensing devices, due to the unique material properties and geometry advantages. The boron or nitrogen doping introduced in the gas phase during deposition promotes p-type or n-type conductivity. With the establishment of the UNCD nanofabrication techniques, more and more nanostructure-based devices are being explored in measuring basic physical and chemical parameters via classic and quantum methods, as exemplified by gas sensors, ultraviolet photodetectors, piezoresistance effect-based devices, biological applications and biosensors, and nitrogen-vacancy color center-based magnetic field quantum sensors. Highlighted finally are some of the remaining challenges and the future outlook in this area.

## 1. Introduction

Diamond is well known for its superior mechanical, electrical, piezoelectric, optical, tribomechanical, and other properties. As the hardest material, diamond is inert and highly compatible biologically and transparent from infrared (IR) to ultraviolet (UV) optically. Moreover, its electrical conductivity can be controlled via the doping technique to change from an insulator to the ultimate semiconductor. In the past, the progress in the production of inexpensive, high-quality diamond thin films has never stopped, and a breakthrough happened in the 1960s [1] with the arrival of a low-pressure, low-temperature chemical vapor deposition (CVD) synthesis method. Since then, synthetic diamond thin films have become commercially available. Although it is almost indistinguishable from gemstone material in property, the thin-film single-crystal diamond (SCD) produced by CVD is currently limited by its size and cost. Hence, the CVD-grown thin-film polycrystalline diamond (PCD) is considered the most affordable and is composed of tiny diamond crystallites fused by atomic-scale non-diamond (usually graphitic) grain boundaries.

**Figure 1:**
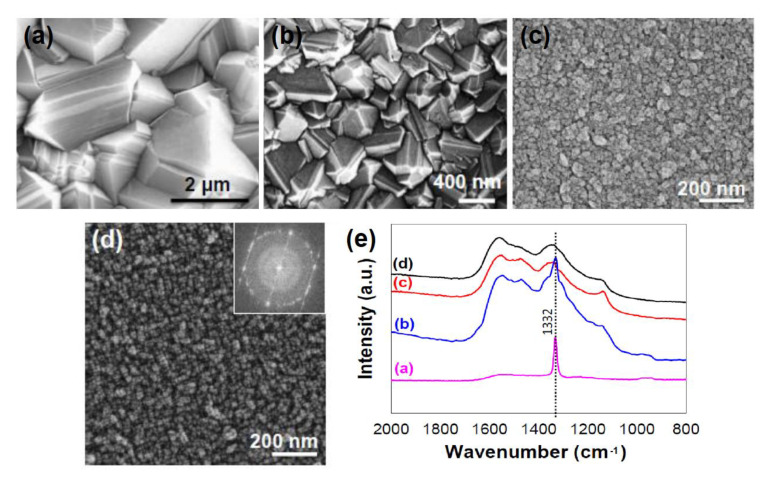
Surface morphologies of different CVD-grown polycrystalline diamond (PCD) films: (**a**) MCD, (**b**) and (**c**) NCD, and (**d**) UNCD. Inset: electron scattering spectra of the UNCD film. (**e**) Raman spectra of MCD (**a**), NCD (**b**,**c**) and UNCD (**d**) films, analyzed with a visible laser beam at 532-nm wavelength. Reprinted with permission from [8]; © 2021, Elsevier.

According to the surface morphologies as shown in Figure 1a–d, polycrystalline diamond films are normally categorized into microcrystalline diamond (MCD), nanocrystalline diamond (NCD), and ultrananocrystalline diamond (UNCD). CVD-grown MCDs are made of relatively large faceted crystallites arranged in different orientations with a grain size of 0.5–100 µm [2,3] and a root-mean-square (RMS) surface roughness typically ~10% of the film thickness, while thin-film NCDs contain many smaller and less facetted crystallites with a grain size in the range of 10–100 nm and surface roughness in the range of 10–50 nm RMS [4]. Invented at Argonne National Laboratory in 2002, UNCDs consist of finest grains of 3–5 nm in size and grain boundary of ~0.4 nm, and they possess excellent thickness uniformity over large area wafers (≥150 mm diameter) and a far smoother surface, with 4–7-nm RMS roughness which is independent of the film thickness [5,6,7,8,9]. Figure 1e shows the Raman spectra of these thin-film materials measured with a visible laser beam at 532-nm wavelength, which indicates that a profound difference occurs at the 1332-cm^−1^ peak.

As a comparison, the properties of CVD-grown SCD and UNCD films are given in Table 1. Unlike SCD, UNCD is widely accepted as the nanoscale composite of ultra-small diamond crystallites with *sp^3^* hybridization surrounded by grain boundaries of a mixture of hydrocarbon and amorphous carbon (a-C), with *sp^2^* character being predominant. However, UNCD still retains sufficient diamond-like properties, which are extremely useful for applications where nanoscale precision machining is needed, due to its finest grain size, small surface roughness, and high surface uniformity, in addition to electrical property modification and surface functionalization. Of all CVD-grown diamond thin films, UNCD is the most promising material platform for the fabrication of well-defined nanowire devices and nano-electro-mechanical systems (NEMS) [10,11,12,13].

Any materials with one or more dimensions that are constrained to the nanometer scale are considered nanomaterials. According to this definition, geometries such as nanotubes, nanorods, nanoribbons, nanofibers, and nanowires exemplify 1D nanomaterials with two dimensions reduced to the nanometer scale. Hence, a structure of a constrained cross-sectional area to tens of nanometers or less and an unconstrained length is considered a nanowire. In this review, we will mainly concentrate on the UNCD NWs which are normally formed through either self-assembling or nanofabrication processes.

Over the past decade, great progress has been made in the fabrication of UNCD nanowires, which has enabled significant developments in diamond-based sensor technology, taken to a radically new level of performance, and brought in new applications. Since there are already a few reviews on other diamond-related materials or self-assembled/as-synthesized diamond NWs [14,15,16], this review focuses mainly on the investigations of various sensors based on UNCD NWs and other nanodevices fabricated using electron-beam lithography and reactive ion etching techniques conducted by our group in recent years.

## 2. UNCD Synthesis and NW Fabrications

### 2.1. Synthesis of UNCD Films

According to the activation energy source, the CVD synthesis technique can be classified as hot-filament CVD (HFCVD) [17], microwave plasma CVD (MPCVD) [18], and radio-frequency CVD (RFCVD) [19], etc. Among several methods of growing high-quality diamond in the laboratory, MPCVD is one such method widely used for the deposition of UNCD films on non-diamond substrates such as Si and SiC [20]. During the early stage of the optimal UNCD growth, the initial surface pretreatment, or “seeding”, is crucial to enhance the nucleation of diamond grains, and the reported seeding techniques include polishing/scratching the substrate using diamond nanoparticles (DNP) [21], ultrasonication of DNP slurry [22], pre-coating and converting of carbon-based material to diamond nuclei [23], and so on. The system contains a microwave generator of up to 2-kW power at 2.45-GHz or 915-MHz frequency. The chamber is maintained at around 100 Torr with a gas mixture of 95–99% Ar, 0–3% of H_2,_ and 1% of CH_4_. Methane is used as the carbon-containing gas due to its high purity, the same structure (tetrahedral) as diamond, and the ease of control of deposition reaction. Driven by the microwave plasmas, the collision between the electrons and gases generates a high fraction of ionized species and therefore provides abundant chemically active ions for UNCD growth. The substrate temperature varies from 400 to 800 °C.

Although UNCD and NCD are two closely related synthetic diamond films, they have distinct growth processes and nanostructures. In NCD growth under hydrogen-rich growth conditions, the hydrogen abstraction takes place to replace each C–H bonding of the CH_4_ with the C–C bonding individually. This process gives the time for the columnar diamond crystal to grow, which leads to higher surface roughness. Unlike NCD, due to its unique growth mechanism under Ar-rich growth conditions, the decomposition of CH_4_ can be described in only two steps as shown below [24]:

(1) $$2CH_{4}\to C_{2}H_{2}+3H_{2}$$

(2) $$C_{2}H_{2}\to C_{2}+H_{2}$$

The carbon dimer (C_2_) radicals can directly insert into the surface of the diamond film due to their low activation energy, which avoids the need for hydrogen abstraction [25]. During the UNCD growth, such a diamond structural formation path is expedited and simplified at a much higher renucleation rate as compared to NCD growth. Therefore, the grain coarsening does not occur, and the continuous growth of the diamond crystal is confined [11]. Compared with NCD, UNCD has a smaller surface roughness, which does not change with the increase in UNCD film thickness. This feature of UNCD films makes it possible to push the dimensions of miniature devices fabricated using the EBL-RIE (Electron-Beam Lithography-Reactive Ion Etching) approach to the limit and to take the advantage of nanowire and NEM devices based on this affordable diamond material for sensor and other applications.

### 2.2. UNCD Doping Techniques

As a natural insulator, the high-resistivity UNCD film can be doped during the synthesis process with nitrogen as a donor or boron as an accepter, to form p- or n-type semiconductors. With the addition of nitrogen (N_2_) in the gas mixture during the synthesis process, nitrogen atoms can incorporate in the grain boundaries and form the nitrogen-doped UNCD (N-UNCD) n-type conductivity in the UNCD film, as demonstrated by Hall and Seebeck’s coefficient measurements [26,27]. As the nitrogen in the gas phase increases to 20% in the reactor, the N-UNCD measured at room temperature (RT) shows a decrease in electrical resistivity down to 10^−2^ Ω·cm, or an increase in conductivity up to 100 Ω^−1^cm^−1^ [28]. Listed below are typical parameters used in the N-UNCD growth process. The MPCVD starts to pump down to 10^−6^ Torr first. The growth takes place for 1 hour in a gas mixture of argon (89%), methane (4%), hydrogen (2%), and nitrogen (5%) under 80-mbar gas pressure with 2000-W plasma power. The synthesized N-UNCD film has a thickness of 100 nm and an RMS surface roughness of 4–7 nm [11]. The conductivity increase in N-UNCD is due to the grain boundary conduction since nitrogen is preferentially incorporated into the grain boundaries. The amount of nitrogen in the grain boundaries rises as the percentage of nitrogen in the plasma increases. In general, the so-called “0%” nitrogen sample might have been doped with N_2_ from the residual nitrogen in a reactor. With a lower base pressure in a reactor, UNCD films could be grown with a conductivity of orders of magnitude less than 0.1 Ω^−1^cm^−1^.

**Figure 2:**
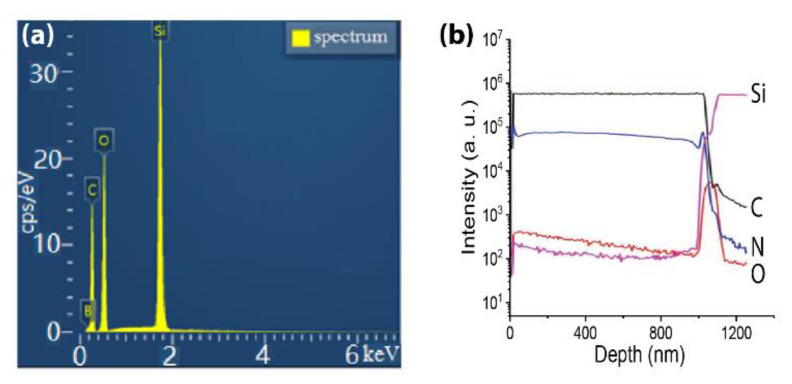
(**a**) EDS spectrum of the B-UNCD NWs, with a B/C atom ratio of 0.227. Reprinted with permission from [32]; © 2021, Elsevier. (**b**) SIMS spectrum from a 1-μm-thick N-UNCD film. Reprinted with permission from [33].

In contrast, by adding diborane (B_2_H_6_) or trimethyl borane (TMB) (B(CH_3_)_3_) during the growth process, the boron-doped UNCD (B-UNCD) can be synthesized as a p-type semiconductor. Unlike the incorporation mechanism of nitrogen doping, the boron doping is substitutional; therefore, the doping level can be very high [29]. When the boron concentration reaches 3 × 10^20^ cm^−3^, the conductivity of the doped diamond film will drastically transform from insulating to metallic. At a temperature lower than 4 K, the superconductivity can be observed [30]. As an example, the energy-dispersive X-ray spectroscopy (EDS) spectrum in Figure 2a shows that the boron level in the doped diamond NWs is around 0.227, expressed as B/C atom ratio. The 100-nm-thick B-UNCD sample is prepared by using HFCVD with a 1-μm SiO_2_ sacrificial layer underneath. As a comparison, the secondary ion mass spectrometer (SIMS) spectrum of an N-UNCD sample is shown in Figure 2b, where the deposited layer has a thickness of approximately 1 μm.

To avoid the possible B contamination of the chamber, post-growth thermal diffusion has been demonstrated for the B-doping of UNCD films [31]. A commercial boron-containing spin-on-dopant (SOD) solution is spin-coated to form a 200-nm-thick film on the UNCD surface. Then, the sample is annealed in the N_2_ environment through a certain temperature profile up to ~1000 °C using a rapid thermal processor (RTP). Although it was reported that the X-ray diffraction (XRD) and Raman spectroscopy showed no evidence of graphitization and structural damage in UNCD films after the thermal diffusion, the boron distribution was not uniform along the UNCD thickness direction. It was also unclear whether this boron doping was substitutional, and how large the stress was, caused by this thermal diffusion process.

Obviously, the capability of both n-type and p-type doping of UNCD, combined with the well-controlled nanostructure fabrication technique described below, enables access to an enormous range of applications using the semiconductor property of diamond, such as field emission (FE)-based miniature devices [34], photovoltaic and energy storage devices [35], water treatment, electrochemical devices [36], biosensors and bioactuators such as nerve stimulating electrodes [37], sensors based on the piezoresistivity effect, and power electronics [38] in extreme environments such as high pressure, high and low temperature, chemical corrosion, and high radiation.

### 2.3. Electron-Beam Lithography (EBL) and Inductively Coupled Plasma Reactive Ion Etching (ICP-RIE)

EBL is the process of writing a pattern using a focused e-beam on a thin organic polymer film called “resist” to change its chemical bonding and thus the solubility in the developer. The focused e-beam writes the smallest features with a resolution in the range of 0.06–0.15 nm, depending on the incident electron energy. After the subsequent development step, the patterned resist film acts as a binary mask for further processing such as reactive ion etching (RIE). The fabrication of more complicated devices may need this procedure more than once to finally engrave the UNCD film into a useful nanoscale device.

With the smallest size reported typically in the range of a few nanometers on bulk silicon (100) and 50-nm-thick silicon nitride membrane, EBL is the standard high-resolution technique to pattern the nanoscale features as designed [39]. The obtained minimum size is ultimately limited by the scattering effect [40] of the electrons, the specific nature of the resist interacting with the high-energy electrons and the material properties to be processed. In general, a 100-keV EBL system is used to pattern the nanoscale structures on UNCD films. Since e-beam lithography transfers a pattern electronically, it permits great flexibility in trying out different patterns on the same batch for a controlled experiment.

After the EBL process, the pattern needs to be converted from the resist layer to the UNCD substrate to make a real device. This transfer process normally takes place top-down by reactive ion etching the UNCD material not covered by the resist, which is also known as plasma etch or dry etch since no wet chemistry is involved. To achieve a high-resolution pattern transfer, the etching process is preferably straight down, which means isotropic etching. To prevent the strong physical sputtering from destroying the pattern layer, an inductive coupling plasma (ICP) is introduced into the RIE system. Therefore, the plasma is generated and controlled separately from the plasma etching.

The standard RIE systems for the UNCD nanoscale device fabrication process use the same equipment employed for silicon (Si) wafers. The following is a UNCD etching recipe to achieve a removal rate of approximately 650 ± 80 nm/min [41]: RIE power = 200 W, ICP power = 2500 W, O_2_ = 50 sccm (cm³/min in standard conditions for temperature and pressure), SF_6_ = 0.5 sccm, pressure = 9 mTorr and temperature = 20 °C. This recipe can be modified for different etch rates. For example, SF_6_ can be replaced with other fluorine-containing gasses such as CF_4_. Above all, ICP-RIE has been routinely used for the high-resolution, high-aspect-ratio etching of UNCD films, removing the etching mask, and releasing NWs from a SiO_2_ substrate.

### 2.4. EBL-ICP-RIE-Based UNCD NW Fabrications

In general, the UNCD NWs can be categized into two groups: self-assembled or as-synthesized NWs, and EBL-RIE processed NWs. According to its orientation, an NW can be aligned either vertically or horizontally. The horizontally aligned UNCD NWs are preferably used for various sensor applications such as gas, UV, and piezoresistivity sensors, while the vertically aligned UNCD NWs are particularly useful for DNA sensing [42], nitrogen-vacancy (NV) color centers [43], and field electron emission [44,45]. Below, we will concentrate on the two most common techniques developed for the fabrication of UNCD nanostructures using the EBL/RIE approach [46]: top-down and bottom-up processing. The former refers to a subtractive process in which UNCD is removed from the top to produce the well-defined nanostructure, while the latter refers to an additive process in which deposition occurs only at defined places to build up the desired nanodevices from the substrate.

#### 2.4.1. Top-Down Fabrication Technique of UNCD Nanostructures 

**Figure 3:**
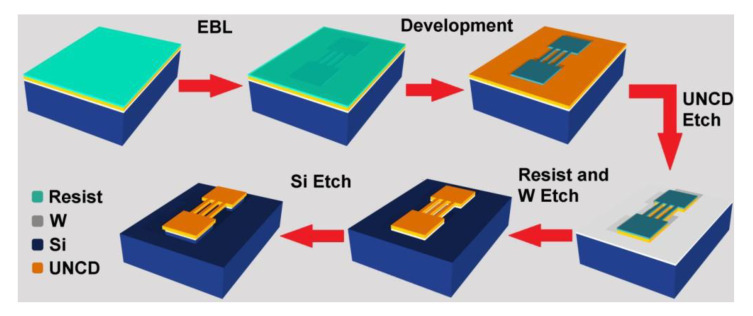
Schematic of the fabrication of UNCD nanostructures via EBL-RIE. Reprinted with permission from [52].

**Figure 4:**
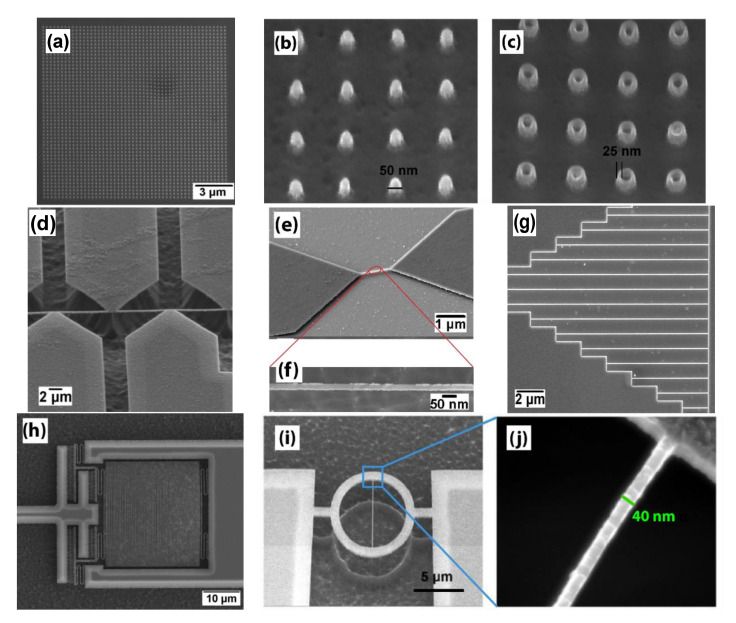
Examples of UNCD nanofabrication. (**a**) Wafer-scale fabrication of UNCD NR array. Tilted scanning electron microscopy (SEM) images of (**b**) UNCD NRs, and (**c**) NTs with 25-nm wall thickness. Reprinted with permission from [46], © 2021, IOP Publishing. (**d**) A long, free-standing UNCD NW supported by multiple pads. (**e**) Single free-standing UNCD NW and (**f**) the magnified SEM image of 25-nm width. (**g**) An array of UNCD NWs of 2 to 18 μm in length. (**h**) Serpent type UNCD NWs separated by a pitch of 200 nm. Reprinted with permission from [52]. (**i**) SEM image of the ring-shaped resonator, and (**j**) A zoom-in view of the resonator shows that the single NW has 40-nm width. Reprinted with permission from [46], © 2021, IOP Publishing.

The top-down fabrication techniques have been successfully used to obtain predefined nanostructures on wafer-sized UNCD thin films [47,48,49]. Figure 3 illustrates the schematic of a typical top-down fabrication process based on EBL and RIE techniques and Figure 4 shows some examples of UNCD NWs and other nanodevices fabricated using this technique. To begin with, a 10-nm-thick tungsten (W) layer is deposited onto the 4-inch-diameter Si substrate as an interlayer to enhance nucleation density [50,51]. The wafer is then seeded with DNPs by immersing into a nanodiamond suspension via ultrasonication. The UNCD films are grown using the MPCVD technique.

Then, the UNCD surface is coated with a layer of hydrogen silsesquioxane (HSQ) to form a negative tone electron beam resist. A 100-nm spin-on-glass layer is formed on the sample by spinning Flowable Oxide (FO_x_) 12, 1:1 diluted by methyl isobutyl ketone (MIBK), at speed of 5000 rpm. Onto the resist, NW patterns are written by a 100-kV EBL system with dosages ranging from 1200 to 1350 μC/cm^2^, depending on the NW widths. The wafer with the exposed resist is then developed in 50 °C MF-CD 26 for 2 min and rinsed in 50 °C deionized (DI) water for 1 min. Finally, two steps of RIE are performed to fabricate UNCD NWs. The NW patterns are firstly transferred from the HSQ mask to the UNCD film underneath by ICP-RIE to create UNCD NWs with typically 1200-W ICP power and 10-W RF power. At an oxygen flow rate of 50 sccm and a chamber pressure of 10 mTorr, an etching rate of ~50 nm/min is achieved. It takes around 2 min to etch UNCD down to the tungsten layer. Finally, the HSQ mask removal and W/Si undercutting are realized by fluoride-based RIE with a typical recipe of 10-sccm sulfur hexafluoride (SF_6_) gas flow rate, 310-W ICP power, 10-W RF power, and 10-mTorr chamber pressure. The release of the NWs from W/Si and cleaning of the HSQ mask thoroughly are accomplished in around 6 min.

As the most common technique for nanoscale patterning, EBL has enabled the fabrication of UNCD NWs, nanorods (NRs), and nanotubes (NTs) with sizes down to tens of nanometer range at a high density. Examples given in Figure 4 include a UNCD NR array of 50 nm in diameter (Figure 4a,b) and an NT array of 25-nm wall thickness (Figure 4c). Figure 4d,e show free-standing UNCD NWs supported by bowtie-shaped pads. It can be seen from the magnified view in Figure 4f that the straight NW has a width as narrow as 30 nm. A UNCD NW array (NWA) of different lengths ranging from 2 to 18 μm has been fabricated on the same batch, as shown in Figure 4g. A dense integration in serpent type of UNCD NWs separated by a gap of 200 nm is given in Figure 4h. Figure 4i,j show a ring-shaped resonator with a single NW of 40 nm in width. The fabrication of these UNCD nanostructures with well-controlled features is essential for sensor and “lab on a chip” applications.

By using EBL to pattern a resist layer as an etch mask followed by the RIE process, various diamond-based nanostructures have also been fabricated, including SCD and PCD NWs, SCD nanorods (NRs), and nanotubes (NTs) [53,54,55]. However, it is still challenging to produce horizontally aligned UNCD NWs with a nanoscale width and a relatively long length without any residual stress. Additionally, the minimum width of diamond NWs has not reached such a level of as small as a few nanometers. As far as the device performance, efficiency, and resolution are concerned, the realization of a smaller dimension and larger surface-to-volume ratio will provide a further improvement, especially for sensors with high spatial resolution.

#### 2.4.2. Bottom-Up Fabrication Technique of UNCD Nanostructures

Although the conventional top-down approach provides well-controlled features, the multiple processing steps often lower the yield and introduce the issues of UNCD contamination and degradation. Therefore, the patterning of UNCD nanostructures from the growth stage has become very attractive. It has been shown that an effective seeding process is important to obtain high-quality UNCD films. An initial nucleation density above 10^11^ cm^−2^ is required to produce a continuous, pinhole-free UNCD thin film [56]. To improve the seeding density, a 5–10-nm-thick tungsten (W) layer is often deposited on a Si substrate first. Such a thin layer attracts nanodiamond seeds evenly during ultrasonication and reduces the initial incubation time. Due to the tungsten carbide formed at the interface, much better uniformity and smaller surface roughness are achieved [57,58]. Since the nucleation process controls the conformity of the UNCD thin film [59], by tuning the seeding density at specific regions, the diamond-based nanostructures can only grow at these predefined locations. To achieve this goal, two major paths have been developed: (1) etching the seeded substrate lithographically to define the pattern, and (2) seeding the patterned substrate. An ink-jet printing method has also been proposed to pattern the seeding layer [60]; however, it is too challenging to pattern a seeding layer of nanosized features.

**Figure 5:**
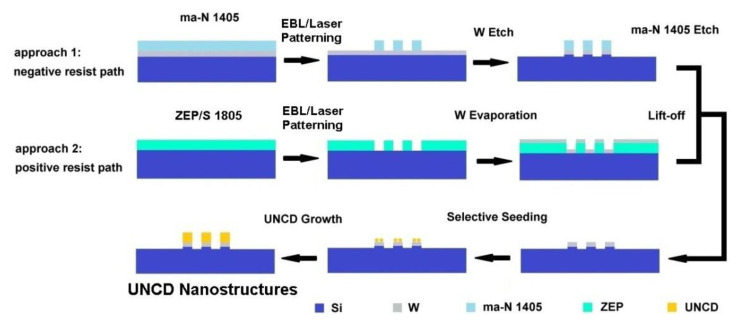
Schematic of bottom-up selective growth of UNCD nanostructures. Reprinted with permission from [46]; © 2021, IOP Publishing.

Two paths have been developed for nanoscale patterning, as shown in Figure 5, by using either positive or negative tone resist. For example, the negative resist path uses the magnetron sputtering system to deposit a 10-nm tungsten layer onto 4- and 6-inch Si wafers. The ma-N 1405 negative resist 1:1 diluted by anisole is then spun onto the tungsten layer at 8000 rpm for 45 s, followed by 90 s 100 °C baking to form a 200-nm-thick resist layer which is patterned with nanoscale features by EBL at a dosage of 900 µC/cm^2^ with a 400-pA current. The etch mask is finally formed after the EBL-exposed resist is developed using ma-D 533/S for 20 s at room temperature, then rinsed with DI water and blow-dried by N_2_. Then, the sample is placed into the ICP-RIE system. The etching of the patterned W layer lasts for around 25 s with 10-sccm SF_6_ gas, 10-W RF power, 310-W ICP power, and 10-mTorr chamber pressure when the sample is kept at a temperature of 20 °C. After the pattern has been successfully transferred from the mask to the W layer, the resist layer is removed by the oxygen plasma RIE in the same chamber for 4 min with 10-W RIE power at 85-mTorr chamber pressure at room temperature. An additional ultrasonic cleaning of the substrate surface is performed with acetone before the seeding process.

During the seeding process, the water-based seeding suspension solution is used. It contains 1% wt, 5-nm “blueseed” DNPs with zeta potential (ζ-potential) of −45 mV, which is 1:4 diluted with DI water. The wafer proceeds with a 10–15-min ultrasonication in the solution, then a 5-min ultrasonication in DI water. After rinsing with DI water for 1 min, the sample is blow-dried by N_2_ and immediately loaded into the MPCVD chamber for UNCD growth. The UNCD film is grown at 760 °C substrate temperature with 2100-Watts microwave power, using Ar/CH_4_/H_2_ gas chemistry with flow rates of 400/1.2/8 sccm, respectively. Throughout the UNCD deposition, the chamber pressure is maintained at 120 mbar. A processing time of 30 min results in a 50-nm-thick film grown on the patterned W area. Due to the high-resolution EBL patterning and selective seeding with a high nucleation density over the 10^11^ cm^−2^ threshold, bottom-up fabrication of UNCD nanodevices has been realized on Si, W [57,58], and SiO_2_ surfaces [61].

**Figure 6:**
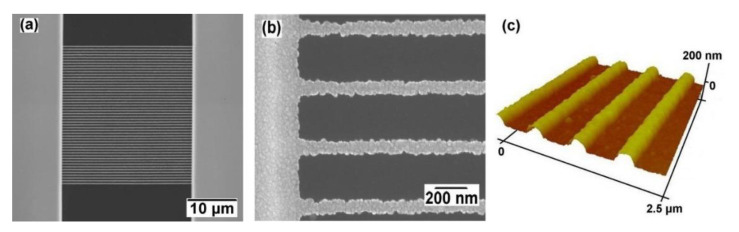
The selectively grown UNCD NW array. (**a**) SEM image of an array of 50 NWs. (**b**) The magnified partial view of (**a**). (**c**) AFM image of (**b**). Reprinted with permission from [46]; © 2021, IOP Publishing.

An example of UNCD NWs grown on the EBL patterned and selectively seeded W layer is shown in Figure 6. The SEM image of an array of 50 NWs suggests high repeatability and consistency of this bottom-up selective growth at nanoscales (Figure 6a). The 90-nm width of NWs measured by the atomic force microscope (AFM) (Figure 6c) is currently limited by the resist resolution.

With the same design, this selectively grown UNCD NW array shows a comparable profile quality with the top-down approach [46]. The density of the NW array is high, with a gap of around 400 nm between two NWs. The distinct UNCD growth with a straight and conformal NW profile in contrast to a clean substrate within such a narrow space suggests the excellent selectivity of the water seeding-based UNCD growth at nanoscale resolution.

By tuning the right combination of e-beam dosage and e-beam resist, nearly 10-nm EBL resolution can be expected [62]. Note that the diameter of nanodiamond seeds is around 10 nm as well; it is thus very exciting to synthesize UNCD NWs of a few nanodiamond seeds in width based on the selective seeding and bottom-up growth method. Since UNCD’s nanocrystals are surrounded by *sp*^2^-bonded grain boundaries, an investigation of the UNCD NWs’ electrical properties at such a scale will provide new insights into the UNCD’s electron transport behavior.

## 3. Characterizations of UNCD NWs

### 3.1. Structural Properties of UNCD NWs

**Figure 7:**
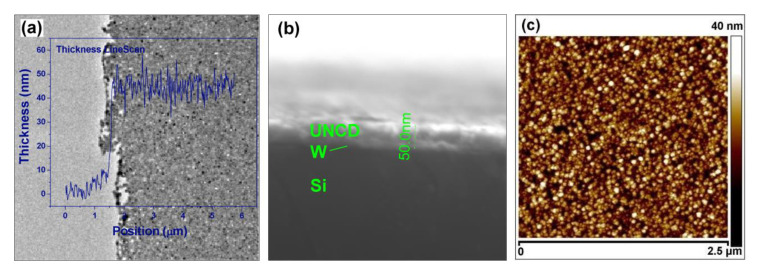
(**a**) TEM image shows the pinhole-free UNCD film. The electron energy loss spectroscopy (EELS) thickness measurement overlaps with TEM, indicating a 50-nm film thickness. (**b**) The cross-sectional SEM image, confirming the 50-nm thickness of the film. (**c**) AFM image of the UNCD film with an RMS surface roughness of 4.5 nm. Reprinted with permission from [52].

UNCD films and UNCD NWs are characterized by various techniques to explore their properties. SEM has been used throughout the film deposition and the NW fabrication process to inspect the quality of diamond films, the control of process flow, and the structure of nanowires. For example, SEM (Figure 1a,b) and high-resolution SEM (HRSEM) (Figure 1c) are used to study the thin-film morphology, to observe the fine diamond grain distributions and grain sizes. Another use of SEM is to determine the UNCD film thickness by the cross-sectional view (Figure 7b). In contrast, AFM has been employed to reveal the detailed surface information (Figure 7c). It is particularly useful to determine the residue level of transparent resist, through the mapped 3D profile of the sample. Another major concern about the diamond nanowire (DNW) fabrication process is the line edge roughness (LER).

Ideally, the fabricated feature follows strictly the design. However, it is determined by both the resolution of the resist mask and the anisotropic etching mechanism. The LER is inspected by AFM, due to its nanometer resolution. As shown in Figure 7c, the AFM image reveals more morphological information of the UNCD NWs. Note that the image is taken on unreleased UNCD NWs because of the scanning limitation of the AFM tip on high-aspect-ratio features; thus, some HSQ e-beam resist residue remains on the top of the NWs. Since the sidewall morphology depends on the UNCD’s surface roughness and grain size, a straight and smooth sidewall profile is formed (Figure 4f), enabling the fabrication of such a small diamond NW as narrow as 25 nm. Another important parameter is the surface roughness, which can be measured using either AFM [63] or white light interferometer (WLI) [64].

**Figure 8:**
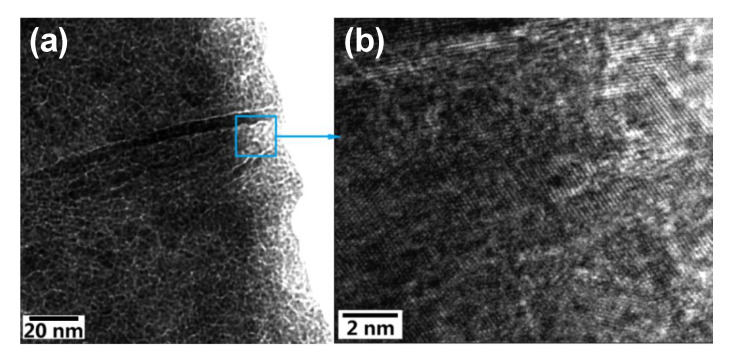
(**a**) TEM image of a UNCD NW whose dark dots represent UNCD grains, and (**b**) its HRTEM image which shows the diamond intrinsic structure is maintained after the etching process. Reprinted with permission from [55].

While SEM and HRSEM provide sample surface images by detecting reflected or knocked-off electrons, transmission electron microscopy (TEM) and high-resolution TEM (HRTEM) use transmitted electrons to enable the inner nanocrystalline structure data of UNCD NWs to be captured. Figure 7a shows the pinhole-free UNCD film, and Figure 8a shows the TEM image taken on top of a UNCD NW, where dark spots represent the diamond nanocrystals and the bright gaps around them are the grain boundaries [65,66]. The HRTEM shown in Figure 8b indicates that no diamond degradation occurred on the edge as diamond nanocrystals distribute uniformly along the NW [52]. As a useful tool to probe possible structural damage, the HRTEM shows that randomly oriented crystalline diamond grains are enclosed by *sp*^2^-bonded grain boundaries. This provides strong evidence of no damage to NW’s intrinsic diamond structure after the chemical etching process.

**Figure 9:**
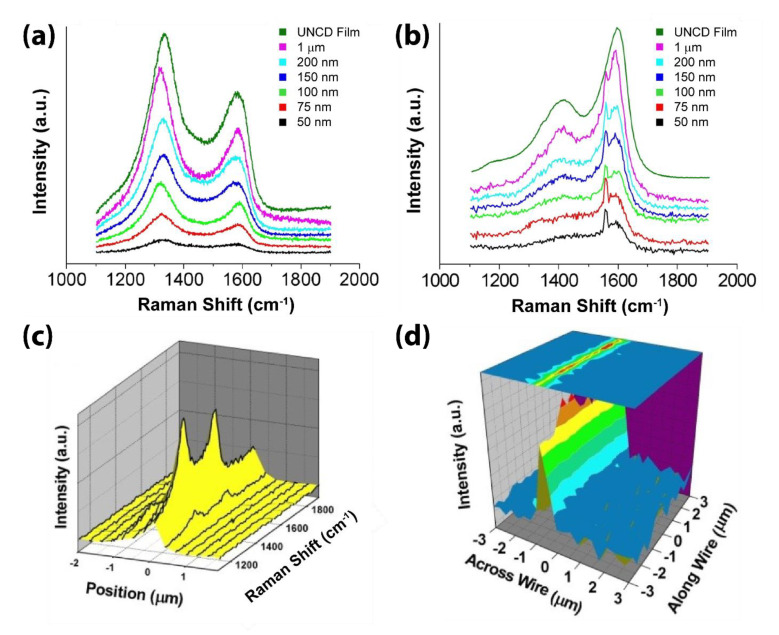
Typical Raman spectra taken on UNCD film and NWs of different widths, (**a**) under 633-nm, and (**b**) 325-nm laser excitation. The peak at 1556 cm^−1^ in (**b**) is attributed to Raman scattering of oxygen gas molecules in the air under UV laser. Under 442-nm laser excitation, (**c**) 1D Raman intensity mapping across a randomly picked UNCD NW, and (**d**) 2D Raman intensity mapping of the peak at 1332-cm^−1^ peak taken at 6 × 6 μm^2^ area across a 6-μm-long NW. Reprinted with permission from [52].

Raman spectroscopy is the most widely used non-destructive chemical analysis technique which provides detailed information about the crystalline quality by discriminating the presence of *sp^2^*- and *sp^3^*-bonded carbon in different bonding configurations that possess different short- and long-range order. When laser beams of different wavelengths are used, due to the well-known resonant Raman effect, a visible laser, with an incident photon energy much lower than the bandgap energy of *sp^3^*-bonded carbon at ~5.5 eV, generates the Raman scattering spectra dominated by *sp^2^*-bonded carbon. Similarly, when UV excitation is used, where the photon energy is shifted closer to the *sp^3^*-bonded carbon, thus it has a much larger Raman scattering cross-section than the *sp^2^*-bonded carbon. To characterize UNCD NW at a global level, confocal Raman microscopy is employed with laser excitations at 325, 442, and 633-nm wavelengths [52], as shown in Figure 9, where the Raman spectra are taken on a randomly picked UNCD film and NWs of different widths. Under 633-nm (Figure 9a) and 325-nm (Figure 9b) laser excitation, each Raman spectrum shows the typical intrinsic UNCD signature, with two broad bands centered at 1332 cm^−1^ from the significant amount of *sp*^3^-bonded carbon and 1595 cm^−1^ from the *sp*^2^-bonded carbon [11]. The spectra profiles from NWs of different widths remain consistent but only display an intensity difference, without peak shift or *sp^2^*/*sp^3^* ratio change. Under 442-nm blue laser excitation, the 1D Raman intensity mapping across an NW is shown in Figure 9c. To further examine the UNCD structure over a broader area, the 2D Raman intensity mapping is performed at a 6 × 6 μm^2^ area with a 300-nm scanning step from the center of UNCD NW. The intensity information at Raman shift 1332 cm^−1^ is extracted from each step’s spectrum, forming the sample intensity distribution map according to the scanning position, as shown in Figure 9d.

**Figure 10:**
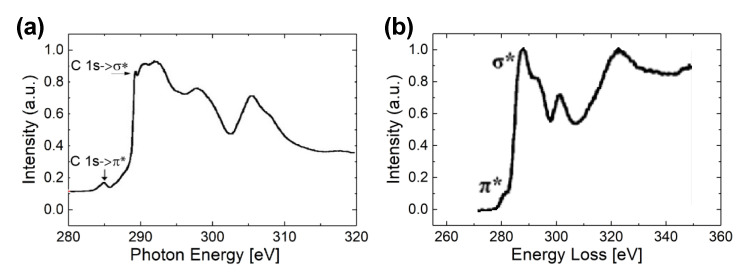
UNCD spectra of (**a**) NEXAFS and (**b**) EELS. The large ratio of carbon σ* to π* peaks suggests a large amount of *sp^3^* bonded carbon (diamond phase) in UNCD NW. Reprinted (**a**) from [53], © 2021, ACS, and (**b**) [52] with permission.

The UNCD composition information can be revealed by using near-edge X-ray absorption fine structure (NEXAFS) spectroscopy and electron energy loss spectroscopy (EELS). The NEXAFS spectrum shown in Figure 10a gives more quantitative chemical bonding information. Nearly 95% *sp*^3^-bonded carbon is calculated from the probing area, which is associated with the σ* peak located at 289.3 eV, whereas for *sp*^2^-bonded carbon which is linked to the π* peak at 285 eV, the fraction is 2.6% [57]. Similarly, EELS taken on UNCD NW is shown in Figure 10b. The high signal ratio of carbon σ* versus π* peaks produces a large amount of *sp*^3^-bonded carbon (diamond phase) in NW, which translates to the high quality of the UNCD crystal structure. The NEXAFS and EELS studies clearly show the presence of a large amount of high-quality diamond grains in the tested samples. As shown in Figure 7a, EELS is also used to verify the film thickness.

**Figure 11:**
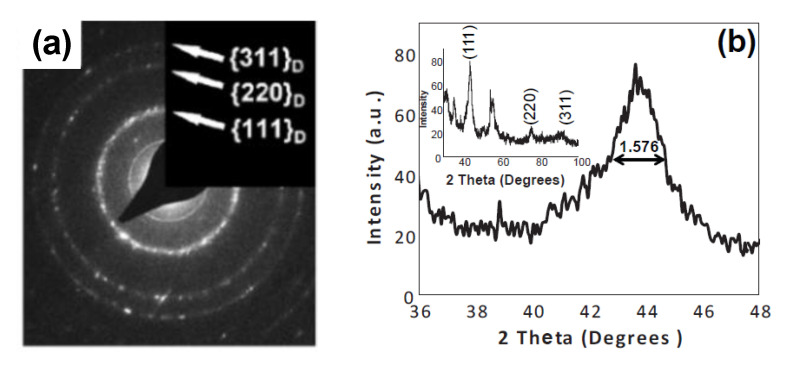
(**a**) SAED pattern of UNCD NW shows the small-sized polycrystalline diamond grain. No significant graphite is identified. Reprinted with permission from [52]. (**b**) XRD spectrum in the region of the diamond (111) peak. The inset shows the whole XRD pattern of the UNCD sample labeled with the diffraction peaks from different planes of the cubic diamond. Reprinted with permission from [8]; © 2021, Elsevier.

The polycrystalline phase of UNCD NW has also been demonstrated by selected area electron diffraction (SAED) in Figure 11a. The results from TEM, Raman spectrum, SAED, and EELS studies conclude that the intrinsic UNCD structure and properties are maintained after the NW fabrication. X-ray diffraction (XRD) is often used to detect crystalline diamond grains. As shown in Figure 11b, the (111), (220), and (311) diamond peaks are clearly discernible, while the other unlabeled peaks are from the tungsten pretreated SiO_2_/Si substrate. According to the measured full width at half maximum (FWHM) of the (111) peak from Figure 11b, the average grain size is in the range of 5.6 nm, estimated from Scherrer’s Equation:

(3) $$L=\frac{K\lambda }{\beta \cos\theta }$$

where *β* = 1.576° is the FWHM, the Bragg angle (2*θ*) equal to 43.65°, the shape factor (*K*) 0.94, and the X-Ray wavelength (λ) 1.5406 Å.

### 3.2. Electrical Properties of UNCD NWs

To explore the possibility of using UNCD NWs as nanoelectronic devices, it is essential to establish their electrical characteristics first. For electrical property measurement, two metal pads made of 10/100 nm of Cr/Au are aligned and patterned on the NW’s supporting pads by a laser patterning and lift-off process. A diluted buffered oxide etch (BOE) is performed afterward to release the NWs from the substrate as well as remove the e-beam resist residue. No critical point drying (CPD) is further needed due to the diamond’s robustness. The electrical measurements are conducted in a SEM/STM (scanning tunneling microscopy) probe station under ultra-high vacuum (UHV) (<10^−9^ Torr). To study the annealing effect, the sample is heated to 150 °C for 5 min within the probe station chamber, then cooled down to RT. Two conducting Platinum/Iridium (Pt/Ir) probes are introduced onto the NW’s metal pads, to provide electrical contact of a DC power supply which is utilized to source the voltage and read current simultaneously. The I-V curve is recorded, with the output voltage ranging from −1.0 to 1.0 V in 0.1-V steps. Throughout the process and measurement, the UNCD NW wafer is kept within the UHV chamber without contacting the atmospheric ambiance.

**Figure 12:**
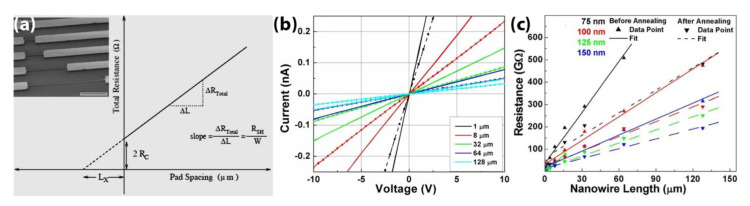
(**a**) Principle of TLM measurement. Inset shows the SEM image of UNCD NWs of different lengths used in the measurement. The scale bar is 20 μm. Measurement results of the N-UNCD NWs before (solid line) and after annealing (dashed line): (**b**) the I-V curves, and (**c**) the TLM curves. Reprinted with permission from [52].

The commonly used transmission line measurement (TLM) [67] is applied to determine UNCD NWs’ electrical properties. The two-terminal current versus voltage (I-V) measurements are conducted in a UHV probe station with the fabricated UNCD NWs of different lengths (1, 8, 32, 64, and 128 μm) and widths (75, 100, 125, and 150 nm), as shown in Figure 12a. As an example, Figure 12b shows the linear I-V plots of a group of 150-nm-wide N-UNCD NWs with different lengths from 1 to 128 μm, corresponding to resistances in the range of giga-ohms (GΩs). As a comparison, after the NWs are annealed at 150 ^o^C for 5 min inside the UHV chamber to remove the possible surface adsorbates of water vapor and gases, the resistances of the UNCD NWs decrease by approximately 30% as, shown in Figure 12b,c.

**Figure 13:**
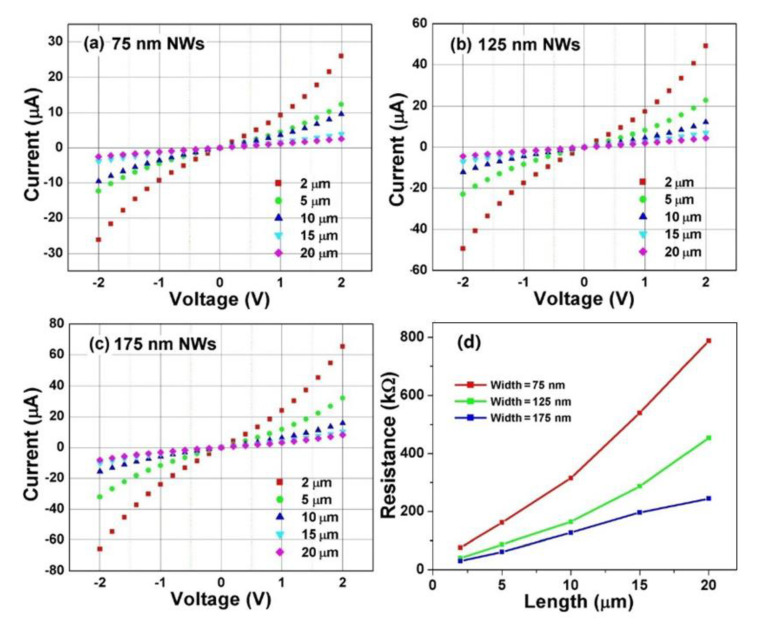
I-V plots of various length B-UNCD NWs with a width of (**a**) 75 nm, (**b**) 125 nm, and (**c**) 175 nm. (**d**) TLM data of the B-UNCD NWs with various lengths and widths. Reprinted with permission from [52].

The TLM characterization has also been applied to boron-doped UNCD NWs. The I-V curves of B-UNCD NWs of 2, 5, 10, 15, and 20 μm long with three different widths of 75, 125, and 175 nm are given in Figure 13a–c, as well as the TLM measurement data in Figure 13d. As shown in Figure 13, the resistances of the B-UNCD NWs are in the kΩ range, around six orders of magnitude lower than the N-UNCD NWs, due to different doping mechanisms. The nitrogen atoms in N-UNCD preferentially incorporate into the grain boundaries, which limits the doping level and the electron transport of the UNCD film [68,69,70,71]. However, the boron atoms in B-UNCD substitute the carbon atoms in the grains, which results in a higher doping capability [72] and much lower material resistance.

After annealing, the resistances of the N-UNCD NWs reduce by 30% at all dimensions. Such an effect is caused by the electron transport dynamics that are influenced by surface adsorption and desorption of gas molecules and water vapor from the environment. For N-UNCD, the nitrogen atoms mostly sit between the UNCD’s crystalline grains; therefore, the electron transport through the grain boundaries forms the conducting network. When the gas and impurities from the atmosphere adsorb on the NW’s surface, they insert into the UNCD’s grain boundaries and thus block the electron transport tunnels and increase the resistance. This effect may not be noticeable in thin-film geometry because the volume is so large that electrons can find more possible pathways. Meanwhile, in NW geometry, because the dimension is drastically reduced and the electron transport tunnel is heavily confined, the surface modification affects significantly the UNCD NW’s electronic property. After annealing, the adsorbates are freed from the NW surface and the electron transport tunnels within the UNCD’s grain boundaries are therefore released and electrons can transfer more freely. The electrical characterization reveals that N-UNCD NWs are a stable ohmic semiconductor with environment-sensitive grain boundaries whose conductivity can be altered by gas/vapor adsorption on the surface [73]. This property makes it possible to build N-UNCD NW-based chemical/biological sensors and NEMS-based integrated multifunctional sensors.

## 4. UNCD NW Sensor Applications

With the tremendous development in UNCD synthesis and UNCD NW fabrication, many UNCD NW-based sensor applications have been explored, including N-UNCD NW-based methane (CH_4_) gas sensors [73,74], B-UNCD NW-based carbon monoxide (CO) gas sensor and sensor arrays [32,75], UV sensors based on UNCD NWs functionalized with platinum nanoparticles (NPs) [76], and piezoresistive B-UNCD NWs. The availability of this special type of DNWs inspires research efforts to make use of both the unique physicochemical properties and geometrical advantages for applications in electrochemical sensors, biosensors, optoelectronics, and nanophotonics. Below are a few research activities conducted recently by our group.

### 4.1. Gas Sensors

#### 4.1.1. CH_4_ Gas Sensors

**Figure 14:**
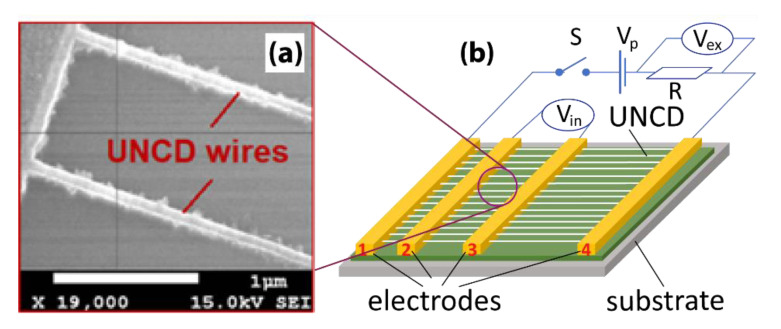
(**a**) SEM image of UNCD NWs between Ti/Au electrodes. The scale bar is 1 μm. (**b**) Schematic of the sensor platform. Reprinted with permission from [73]; © 2021, AIP Publishing.

Since CH_4_ is odorless and colorless, it is important to detect the presence of this extremely flammable and explosive gas. It is also considered as a contributing factor to enhancing the greenhouse effect by absorbing IR. The demonstrated CH_4_ gas sensors use N-UNCD NWs fabricated by the top-down technique. First, 10/100-nm Ti/Au layers are deposited to form the four electrodes, as shown in Figure 14a,b, where the sensing NWs of a width of 150 nm are divided into three areas with four electrodes which form 5, 10, and 20-μm-long NWs between adjacent electrodes. The conductive electrodes “1” and “4” (the external electrode pair) are connected to a switch, a DC power supply with a step voltage *V*_p_, and a high-precision resistor R in series (Figure 14b).

**Figure 15:**
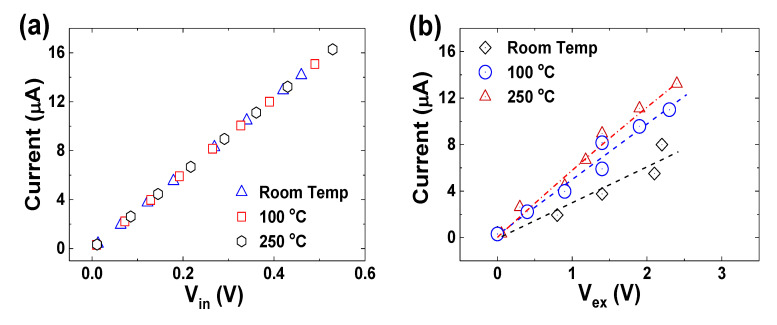
Electrical properties of UNCD NWs characterized at different temperatures by using (**a**) an internal pair and (**b**) external pair of electrodes. Reprinted with permission from [74]; © 2021, IAAM-VBRI.

One voltmeter (*V*_in_) is connected to the electrodes “2” and “3” (the internal electrode pair) and the other voltmeter (*V*_ex_) is connected in parallel with the resistor in the external electrode circuit. Both voltmeters monitor the voltage variations of the gas sensors formed between the internal electrode pair and the external electrode pair simultaneously. The I-V plots of the UNCD NWs at different temperatures are shown in Figure 15, which indicates a resistance of 33 kΩ between the internal electrodes regardless of the temperature (Figure 15a), 350 kΩ at RT, 219 kΩ at 100 °C, and 188 kΩ at 250 °C between the external electrodes (Figure 15b).

The sensor’s electrical properties are affected by the adsorption and desorption of the gaseous molecules. As a result of CH_4_ adsorption, negative charge carriers are added to the material, and hence the resistance decreases [77]:

(4) $${\mathrm{CH}}_{4}\;+\;4O_{\mathrm{adsorption}}^{-}\;\to \;{\mathrm{CO}}_{2\left(\mathrm{air}\right)}\;+\;2H_{2}O\;+\;4e^{-}.$$

Therefore, we can detect the CH_4_ gas concentration by measuring the conductivity change of the sensor. 

From the measurement of the variation of voltage (*V_ex_*) across the precision resistor *R_p_* = 1.0 kΩ, the resistance *R* of the sensor can be obtained by

*R* = (*V_p_ − V_ex_*)*R_p_/V_ex_* (5)

where the power supply voltage *V_p_* = 12 V. Necessary calibrations of the sensor are conducted at the characterization chamber [78]. The response is calculated as

**Figure 16:**
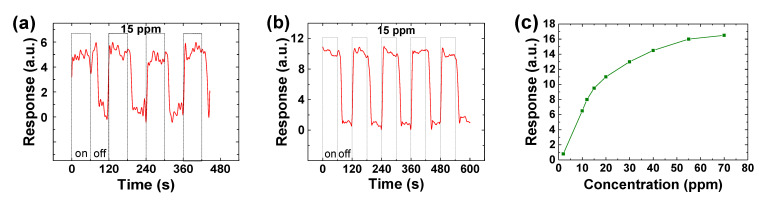
Typical RT responsivities of the N-UNCD NW CH_4_ gas sensor to the “on” and “off” period of the methane gas at the concentration of 15 ppm from (**a**) the internal pair, and (**b**) the external pair of electrodes. (**c**) Responsivities from the external pair of electrodes to methane down to 2 ppm. Reprinted with permission from [74]; © 2021, IAAM-VBRI.

**Figure 17:**
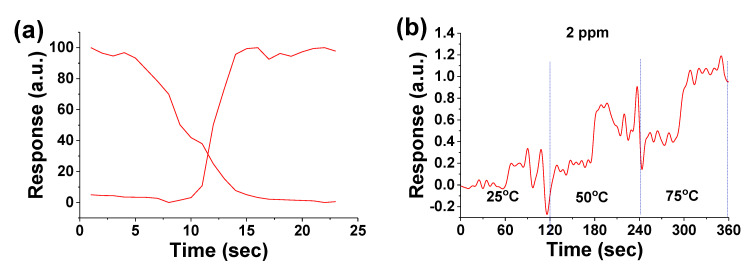
(**a**) The measured sensor response and recovery time of 3 and 9 s. (**b**) The sensor response to methane gas at concentration of 2 ppm when operated at 25, 50, and 75 °C. Reprinted with permission from [74]; © 2021, IAAM-VBRI.

*S* = (*R_air_ − R_gas_*) × 100%/*R_air_* (6)

where *R_gas_* is the sensor resistance measured in the presence of target gas, and *R_air_* is the initial sensor resistance in the air. The typical sensor responsivity to the on-off period of the methane gas of 15 parts per million (ppm) is shown in Figure 16a,b, with a minimum detectable CH_4_ concentration of 2 ppm (Figure 16c and Figure 17b). The sensor shows good repeatability and stability, with a response and recovery time in the order of a few seconds. It is noted that the external electrode pair gives higher responsivity than the internal electrode pair because of the larger exposed sensing area.

The response and recovery time determined from Figure 17a are 3 and 9 s, respectively, which are based on the time duration from 10% to 90% of the full response of the sensor, or vice versa. The obtained response and recovery time are much shorter than the reported values from regular sensors in the range of ~100 s for the response time and >200 s for the recovery time [79]. Figure 17b shows the temperature effect on the sensor responses when exposed to the targeted gas. The sensor output signal increases when the sensor operating temperature is increased from 25 to 75 °C.

#### 4.1.2. CO Gas Sensors

**Figure 18:**
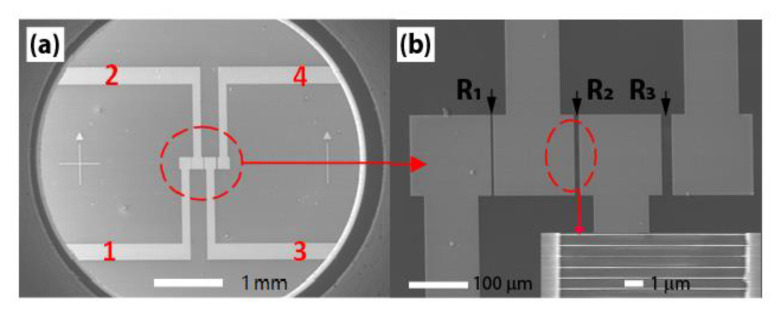
(**a**) Field emission scanning electron microscopy (FESEM) image of the prototypic sensor. (**b**) The four sputtered Au electrodes between 5, 10, and 20-μm-long B-UNCD NWs of resistances of R_1_, R_2_, and R_3_. Inset shows the partial enlargement of the array of 70-nm-wide NWs between two Au electrodes. Reprinted with permission from [32]; © 2021, Elsevier.

CO is an invisible, tasteless, and odorless but serious toxic and flammable gas. Such a sensor is needed to detect this poisonous gas. The CO gas sensors are developed by making use of top-down fabricated B-UNCD NWAs on a 2 × 2 inch Si substrate. The UNCD NWs of 70-nm thickness and 100-nm width are levitated, not in contact with the Si substrate. Figure 18 shows the three groups of NWAs formed between four sputtered Au electrodes with lengths of 5, 10, and 20 μm, corresponding to resistances of R_1_, R_2_, and R_3_, respectively.

**Figure 19:**
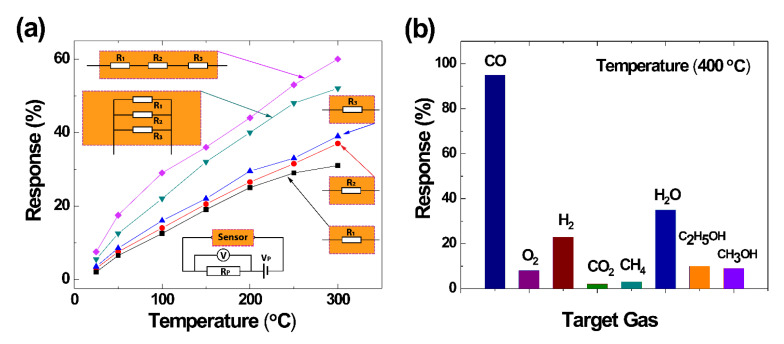
(**a**) Responses of B-UNCD NW arrays to 50-ppm CO gas at different temperatures. Inset: Schematic of the sensor circuit, and the five sensor configurations tested: R_1_, R_2_, R_3_, all in series and all in parallel. Reprinted with permission from [75]; © 2021, Elsevier. (**b**) Responses of the 10-µm-long NW arrays to different target gases with a concentration of 100 ppm, when tested at 400 °C. Reprinted with permission from [32]; © 2021, Elsevier.

The sensing properties are evaluated toward the CO mixed with air at a concentration from 25 to 100 ppm, and the operating temperature from RT to 400 °C. The responses of the sensor arrays, defined by Equation (6), are measured when the NWAs are connected in five different configurations (Figure 19a). The experimental results indicate that the sensor has a high response to CO with good selectivity in this experimental condition, as indicated by R_2_ (10-μm-long B-UNCD NWs) responses to eight different gases of 100 ppm concentration at 400 °C (Figure 19b).

### 4.2. UV Photodetectors

UV sensors of different device structures such as PIN [80], Schottky [81], metal–semiconductor–metal (MSM) [82], and field emission [83] have been demonstrated using synthetic diamond thin films of different morphologies, including single-crystalline diamond (SCD) [82,84,85,86,87,88], microcrystalline diamond (MCD) [83], sulfur-doped sub-microcrystalline diamond (S-SMCD) [83], poly-crystalline diamond (PCD) [89], and nanocrystalline diamond (NCD) [90]. However, their performances were limited by the fundamental issue in electrical transport featured by the high carrier concentration with low mobility in diamond materials. Given the combination of the NW confinement with the nanoplasmonic effect of metal nanoparticles, this new type of UV PDs has shown performance parameters which are comparable with similar devices made of other materials.

**Figure 20:**
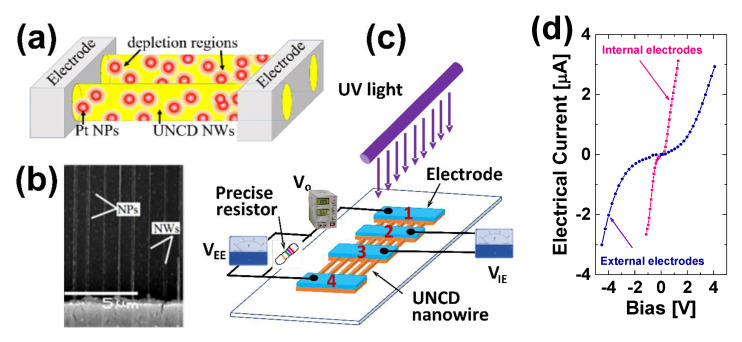
(**a**) Schematic of the B-UNCD NWs functionalized with Pt NPs. (**b**) SEM image of the Pt NP-coated B-UNCD NWs. (**c**) Schematic of the UV PD with four electrodes and the electric circuit. (**d**) The I-V electrical property of the UV PD prototype. Reprinted with permission from [76]; © 2021, ACS.

The materials used in this study are 100-nm-thick B-UNCD films on Si substrates with 1-µm SiO_2_ sacrificial layers, synthesized using HFCVD. The top-down technique is used to fabricate the UNCD NW arrays, followed by the functionalization with Pt NPs in a plasma sputtering chamber (Figure 20a,b). The four electrodes of 10/100-nm titanium/gold (Ti/Au) are deposited on the fabricated UNCD NWs, as shown in Figure 20c, similar to the gas sensors as described above. This electrode design makes use of the four-point probe measurement of sensing performance that efficiently minimizes the polarization effect, possible carrier trapping, and space charges [91,92]. The electrodes are spaced 5, 10, and 20 μm apart in sequence, and there are 110 nanowires between each electrode pair. Each NW has a width of 70 nm, and the gap between any two adjacent nanowires is 1 μm (Figure 20b). The UV PD consists of 330 UNCD NWs between four electrodes and a total sensing area of 270 μm^2^ between the pair of external electrodes and 77 μm^2^ between the pair of internal electrodes. After fabrication, the sensor is annealed at 150 °C for 5 minutes in the probe station chamber. The I-V plots using either an internal or external pair of electrodes are nonlinear, as shown in Figure 20d.

**Figure 21:**
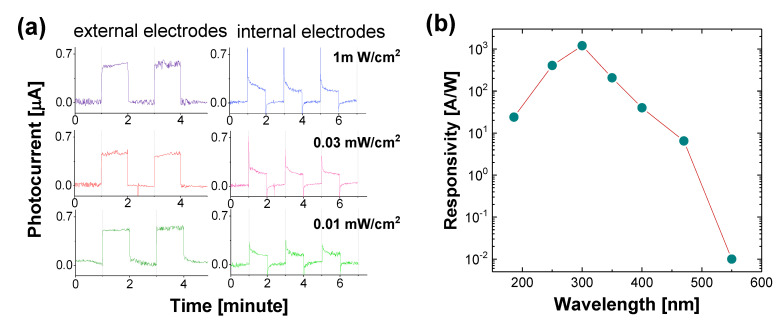
(**a**) Time-dependent photocurrent from the external and internal electrodes at 0-V bias when illuminated by 350-nm light at 0.01, 0.03, and 1 mW/cm^2^, and (**b**) PD spectral responsivity from external electrodes at 0-V bias when exposed to different wavelengths of 1-mW/cm^2^ intensity. Reprinted with permission from [76]; © 2021, ACS.

When exposed to 350-nm UV at an intensity of 0.01, 0.03, and 1 mW/cm^2^, the quick and well-defined responses of external and internal electrode-based PDs under zero bias voltage are observed (Figure 21a). The spectral response to wavelengths from 186 to 550 nm peaks at 300 nm (Figure 21b), indicating that the direct band-to-band transition (~5.5 eV) within the diamond bandgap is reduced in B-UNCD to 4.1 eV based on Mendoza’s model and the relationships shown in Figure 21b, due to the shift of the scattering cross-section caused by the Pt NPs, increment in midgap states corresponding to boron doping, the presence of *sp^2^*-bonded carbon in grain boundaries, and B_3_O and B_4_O defects [83,93,94,95,96], although further investigation of the observed bandgap shift is needed.

The response decreases rapidly at longer visible wavelengths, and the UV-Visible rejection ratio *R*_300_/*R*_550_ goes up by five orders of magnitude. The PD responsivities *R_λ_*, defined as

(7) $$R_{\lambda }=\frac{I_{\lambda }}{W_{\lambda }}$$

where *I_λ_* is the induced photocurrent and *W_λ_* is the incident light power on the sensor surface, which is 207 and 388 A/W at 300 nm from the pairs of external and internal electrodes, respectively.

**Figure 22:**
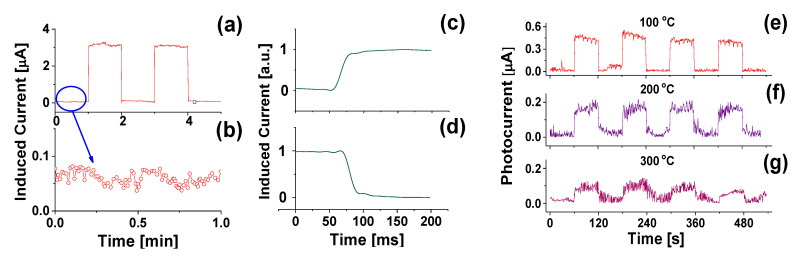
The induced photocurrent (**a**) from the external electrodes when exposed to square-shaped temporal distribution of 300-nm light at zero bias, (**b**) when the UV is off, (**c**) of a typical rising edge, and (**d**) a falling edge. The photoresponse of the UV photodetector when exposed to 250-nm light at 0.03 mW/cm^2^ with 0-V bias at different temperatures from (**e**) 100 to (**f**) 200 and (**g**) 300 °C. Reprinted with permission from [76]; © 2021, ACS.

The dark current, as shown in Figure 22a,b, is ~0.06 µA, whereas the induced photocurrent is ~3 µA, leading to a signal-to-dark current ratio of 50. The fast response and recovery times, defined as the duration taken for the amplitude of a pulse to increase from 10% to 90% of the maximum value, or vice versa, are around 20 ms (Figure 21c,d). The B-UNCD NW-based PD maintains excellent repeatability and stability as the operating temperature is increased up to 300 °C, with a decrease in photocurrent and an increase in thermal noise (Figure 22e–g). This is a considerable improvement in heat tolerance due to diamond’s intrinsic properties, compared to the majority of UV sensors reported.

The reported performance parameters of state-of-the-art diamond-based UV PDs are listed in Table 2. Compared with other devices in Table 2, the B-UNCD NW-based UV PD functionalized by Pt NPs has the highest photoresponsivity of all. Its rise and decay times are fast, in the order of milliseconds, while the dark current level and UV to visible rejection ratio are better than most of the devices reported previously. Furthermore, this prototype is capable of zero-biased and high-temperature operation.

### 4.3. Piezoresistance (PZR) Effect-Based Sensors

The term piezoresistivity/piezoresistance (PZR) effect describes the material resistance change as the result of the modification of its electrical properties, such as bandgap, carrier mobility, and so on, when a strain/stress is built up. For example, Si NWs have shown promising potential in utilizing the PZR effect. However, the strong surface oxidation and the limitation of operating temperature are two major problems [103,104]. Due to the excellent intrinsic properties, especially the high thermal conductivity, diamond-based nanoelectronic devices are excellent candidates for developing the next generation of high-temperature PZR sensors [105].

**Figure 23:**
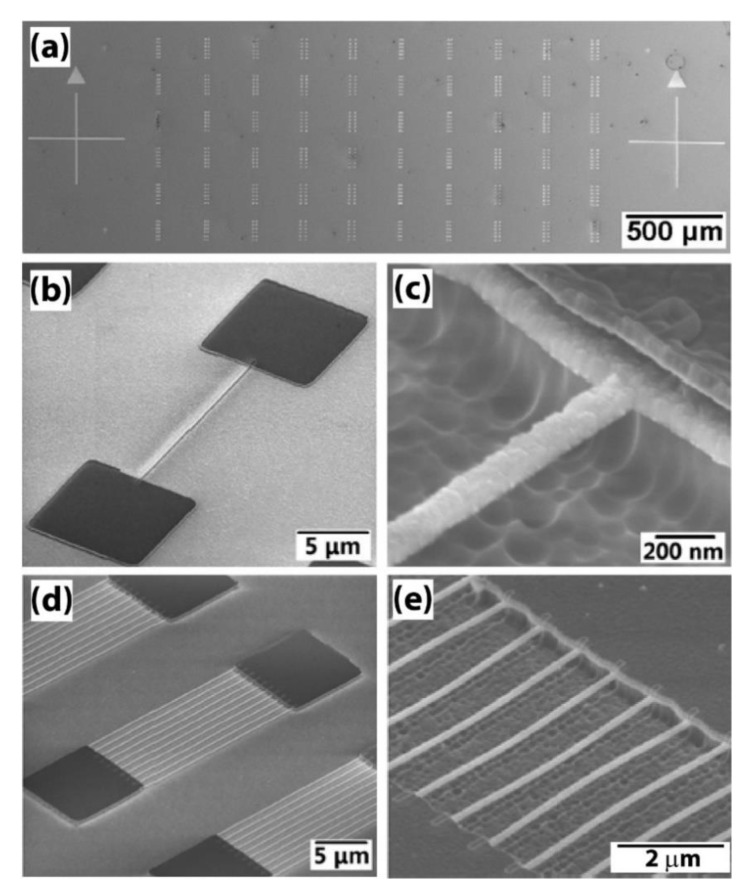
(**a**) SEM image of the 10 × 6 matrix of B-UNCD NW arrays. (**b**) HSQ NW pattern between Ti/Pt metal pads after EBL. (**c**) Released B-UNCD NW after SiO_2_/Si etch. (**d**) HSQ NW patterns between Ti/Pt metal pads after EBL. (**e**) Released B-UNCD NW array after SiO_2_/Si etch. Reprinted with permission from [52].

**Figure 24:**
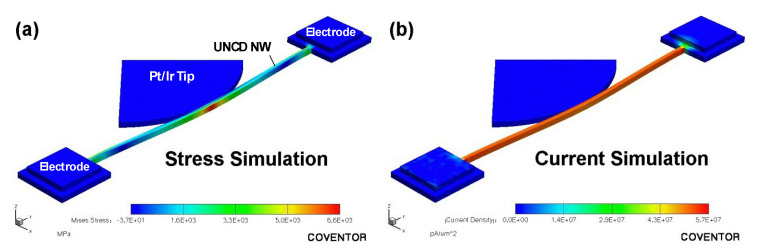
Simulation of (**a**) stress and (**b**) current of the 75-nm-wide and 5-µm-long B-UNCD NW under 100-nm transverse displacement. Reprinted with permission from [52].

The fabrication of the electrode-coated B-UNCD NWs (Figure 23) follows the similar process flow as described before (Figure 23b,d), except there is a 1-μm-thick SiO_2_ sacrificial layer between the 100-nm-thick B-UNCD film and Si substrate, which is finally etched away so B-UNCD NWs are eventually released from the substrate, as shown in Figure 23c–e. The electrical and PZR measurements of the NWs are performed in the SEM/STM probe station under UHV. The PZR effect is measured by using an electrically floating Pt/Ir tip to push the NW of both ends fixed, as shown in the schematic in Figure 24.

Using the remote-controlled box, the tip position is controlled with a spatial resolution of 50 nm. By pushing an NW transversely, a deflection is generated, and the straight NW turns to a curved one. This deflection results in the longitudinal tensile stress of the NW. Figure 24a,b show the stress and the electrical current density distribution simulated based on finite element analysis (FEA) for a 75-nm-wide and 5-µm-long B-UNCD NW under 100-nm transverse displacement. As expected, the highest stress occurs at the contacting points and both anchors, while the current density distributes uniformly along the NW.

**Figure 25:**
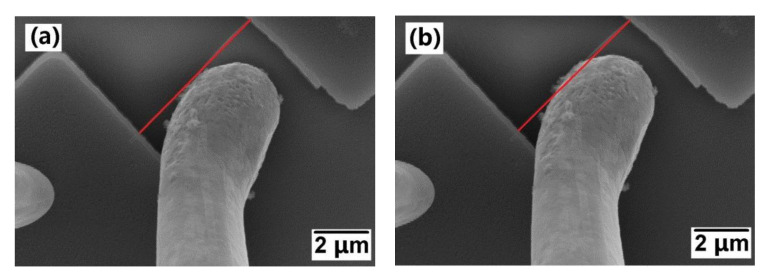
SEM images of (**a**) the unstrained and (**b**) strained B-UNCD NW. The red line indicates the original position of unstrained NW. Reprinted with permission from [52].

A noticeable NW deformation can be clearly observed in Figure 25b, as compared with the red line, which is the original position of unstrained NW (Figure 25a). The standard I-V is measured simultaneously while the NW is pushed by the tip. The sample is also heated to 100 °C to study the PZR effect at different temperatures.

**Figure 26:**
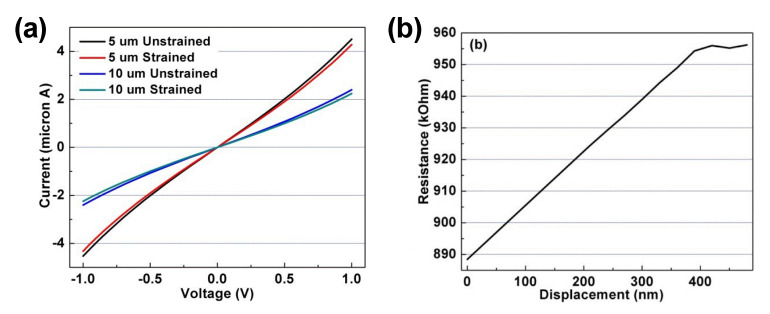
(**a**) The I-V curves of 75-nm-wide, 5 and 10-µm-long B-UNCD NWs with and without strain. (**b**) The resistance of the 75-nm-wide, 20-µm-long B-UNCD NW as a function of the transverse displacement. The NW reaches its damage threshold when the displacement exceeds 400 nm. Reprinted with permission from [52].

Under tensile stress, 75-nm-wide NWs of two lengths (5 and 10 µm) demonstrate the resistance increase, as indicated by the smaller slopes of the I-V curves with strains (Figure 26a). This result is completely opposite to the PZR effect of Si NW with a lower resistance under tensile stress. The symmetric but slightly nonlinear I-V plots agree with the I-V measurements, as shown in Figure 13a. The correlation between the resistance and transverse displacement up to 450 nm is plotted in Figure 26b, which indicates the linear increase in resistance with the transverse displacement until the NW physical damage occurs beyond 400-nm transverse displacement. The results are reproducible below the damage threshold, which suggests that within a certain force range, the B-UNCD NW can be used as a reliable, high-resolution, and ultrasensitive force/pressure sensor.

**Figure 27:**
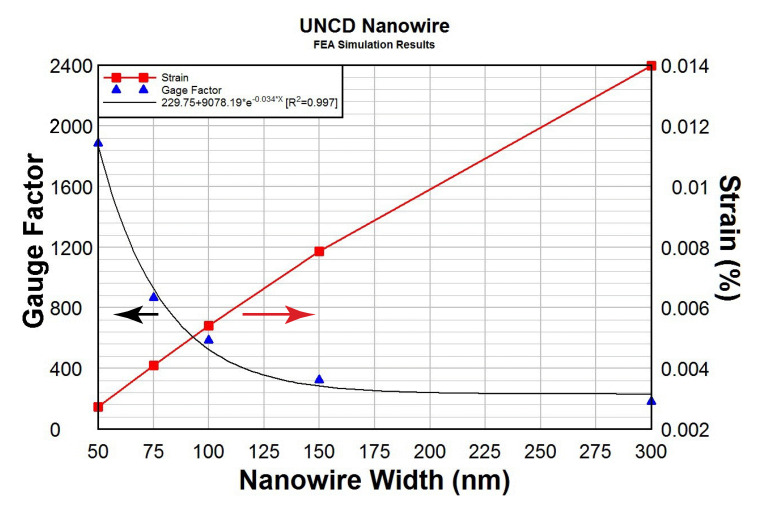
The gauge factor and strain as a function of the width of NW of 5-µm-long by 100-nm-thick. Reprinted with permission from [52].

To describe the sensitivity of the PZR effect, the gauge factor *K* is used [106], which describes the changing sensitivity of material given a certain strain,

(8) $$K=\frac{\left(\frac{dR}{R_{0}}\right)}{d\epsilon }=1+2\nu_{f}+\frac{d\rho }{\rho_{0}d\epsilon }$$

where *ρ_0_* is the resistivity and *ν_f_* = 0.1 is the Poisson ratio for diamond. The strain dϵ can be calculated as:

(9) $$d\epsilon =\frac{dL}{L}=\frac{2\times \sqrt{{\left(L/2\right)}^{2}+D^{2}}-L}{L}$$

where *L* is the NW length and *D* is the transverse displacement generated by the tip’s push. For example, for the 75-nm-wide NW, *L* = 5 µm, *D* = 100 nm, d*R*/*R*_0_ = 5.6%, the calculated gauge factors *K* = 70, which is comparable with the best available Si nanowire results reported so far and are 10 folds higher than nanocrystalline diamond and UNCD films. As expected, the longer the NW length, the higher the gauge factor. Figure 27 shows the FEA simulation of the gauge factor as a function of NW width under different strain levels. When the NW width scales down to 50 nm, the gauge factor of the B-UNCD NW can be as high as 1800, given a strain as small as 0.011%.

The B-UNCD NWs are used for the first PZR measurement because of their smaller resistances as compared with N-UNCD NWs. Its resistance increases linearly with the applied strain, originated from the crystalline structure of B-UNCD nanowires. As indicated, thinner, longer, and narrower B-UNCD NWs of larger PZR effects are excellent candidates for ultrasensitive pressure and force sensors.

### 4.4. Biosensors and Nitrogen-Vacancy Quantum Sensors

Given the combination of diamond’s biocompatibility and bioinertness with its semiconductor property and established nanofab techniques, CVD-grown UNCD thin films and NWs have become the most affordable and preferred material platform for diamond-based biosensors. In general, undoped UNCD can be used for fluorescence detection, while doped UNCD is suitable for diagnoses based on amperometric, gravimetric, potentiometric, resistive/conductive, capacitive, piezoresistive, field emission, magnetometric, and electrochemiluminescence transduction mechanisms. Overall, these UNCD-related biosensors can be normally categorized into two groups: in vivo and in vitro. In vivo biosensors are surface or implant devices that measure one or a range of parameters directly on the living tissue. These include the use as coatings for implants and prostheses in cardiovascular, neural, muscular, epidermal, orthopedic applications [107]. The UNCD surfaced electrodes have been widely applied in electrochemistry [108], sensing the electrical signals from organs or muscles inside the body. The UNCD-coated bio-NEMS are implanted to replace non-biocompatible piezo-actuated bio-NEMS and are implanted in the human retina to restore damaged vision. Due to their mechanical strengths and flexibilities, UNCD thin films and UNCD NWs can also be used in wearable sensor devices.

**Figure 28:**
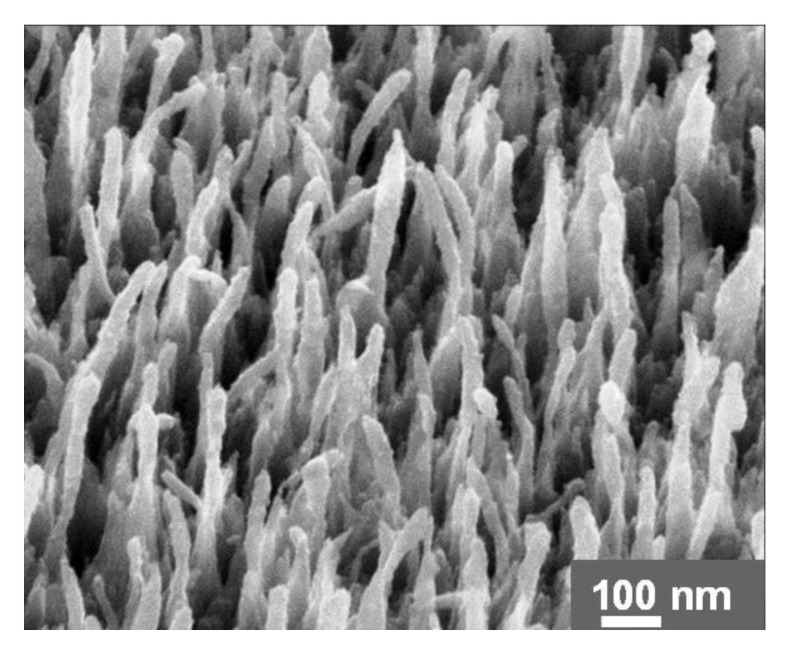
Self-assembled and vertically aligned UNCD NRs. Reprinted with permission from [112]; © 2021, CC BY 4.0.

In vitro biosensors use UNCD as a substrate upon which a range of ongoing biochemical reactions is sensed in a culture dish, a test tube, a microtiter plate. For example, UNCD provides the strongest bonding stability to deoxyribonucleic acid (DNA) [42,109,110] in DNA sensors, and simple and quick detection of viruses [111]. The self-assembled/as-synthesized and vertically aligned UNCD NRs (Figure 28) [112,113] might be advantageous for this type of application; higher sensitivity and better selectivity could be achieved due to the large surface-to-volume ratio.

The most prominent luminescent nitrogen-vacancy (NV) color center in diamond has recently been attracting increasing interest [114,115]. As a unique platform for quantum information technology (QIT)-related components, quantum memories, quantum repeaters [116], and single photon sources [117,118] have been demonstrated. NV color centers, served as tiny quantum magnets with different spins due to the special electronic configuration, convert the received nuclear magnetic resonance (NMR) signals from nearby atoms into visible photoluminescence [119,120]. The room-temperature optical sensing of magnetic resonance using a single NV center has been demonstrated to obtain information about the magnetic properties of individual atoms.

**Figure 29:**
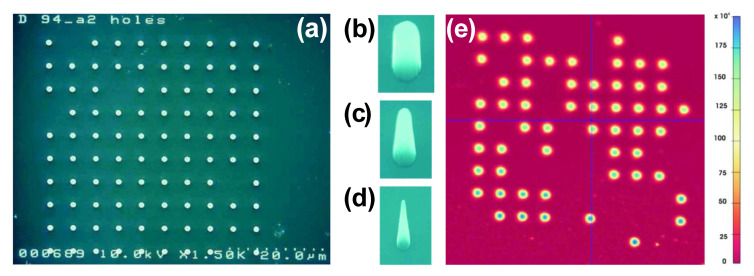
(**a**) UNCD pillar array fabricated using the EBL-ICP-RIE method. (**b**–**d**) Pillars with nominal diameters of 1000, 500, and 200 nm, respectively. (**e**) NV fluorescence mapping of 1000-nm UNCD pillar array. Reprinted with permission from [122]; © 2021, John Wiley and Sons.

NV-based quantum sensors have huge potential in RT detection of magnetic fields, electric field, strain, with nanoscale resolutions [121], and in vivo analysis of the structures of individual proteins and other biomolecules inside cells. It has been reported that UNCD pillars have been fabricated (Figure 29) using the EBL-ICP-RIE method for the investigation of NV centers. The fluorescence mapping and photoluminescence measurements have shown higher photon intensities compared with NCD counterparts [122]. We believe the observed high intensity is due to the nitrogen location in UNCD, the large surface-to-volume ratio, and the light trapping in guided modes of the processed nanopillars.

## 5. Discussion and Outlook

The advancement of the UNCD material infused with nanofab technology has resulted in the development of UNCD-based nanosensors. In addition, new physical phenomena at the nanometer scale have inspired the ultimate potential for these sensors to reach a single molecule or atom sensitivities. When the active sensing area goes down to the nanoscale, device characterization and optimization become more and more important. To optimize electronic transport, device geometry needs to be carefully designed. The Debye length *λ_DL_* and mean free path *λ_MFP_* are two material-related important parameters, given by [123]

(10) $$\lambda_{DL}=\sqrt{\frac{\varepsilon_{r}\varepsilon_{0}k_{B}T}{q^{2}N_{D}}},$$

where *ε_r_* is the dielectric constant, *ε_o_* = 8.85 *×* 10^−12^ C^2^/(N m^2^) the permittivity of free space, *k_B_* = 1.38 *×* 10^−23^ J/K the Boltzmann constant, *T* the temperature in Kelvin, *q* = 1.6 × 10^−19^ C the elementary charge, *N_D_* the net density of dopants, and

*λ_MFP_* = *υτ_m_* (11)

where *υ* is the average drift velocity of charge carriers and *τ_m_* the momentum relaxation time. For B-UNCD samples used for UV sensor applications, the typical values of these physical parameters are *T* ≈ 300 K (RT), *ε_r_* ≈ 5.6, *N_D_* ≈ 2.4×10^18^ cm^−3^, *υ* ≈ 10^7^ cm/s, and *τ_m_* ≈ 50 × 10^−15^ sec [124]. According to Equations (10) and (11), the estimated *λ_DL_* ≈ 0.5 nm, and *λ_MFP_* ≈ 5 nm. Both are on the order of just a few nanometers or less. Hence, the optimal device dimension needs to be further downsized to a few nanometers because only the charge carriers within the Debye length contribute to the conductivity change. Otherwise, the electron and hole recombine before they are collected by the metal contacts. The sensor performance will be further improved if the NW’s width gets smaller.

**Figure 30:**
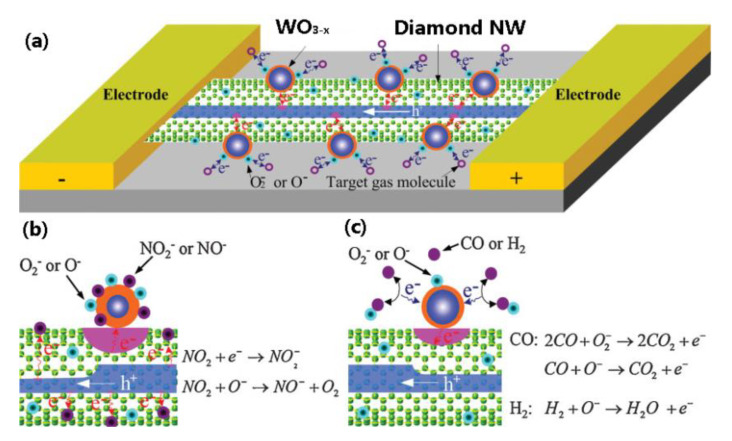
(**a**) Schematic of a hybrid sensing platform. (**b**) The electron transfer between oxidizing gas molecules and the gas sensor. (**c**) The electron transfer between reducing gas molecules and oxygen adsorbates on the gas sensor.

The specific top-down and bottom-up techniques summarized in this review article have demonstrated beautifully the fabrication of various UNCD nanoarchitectures, which provides a possible solution for the integration of a larger number of sensors into a tiny, inexpensive, multifunctional, highly sensitive, and large-throughput sensor array. Since each UNCD NW-based device is small, different sensors can be integrated on the same wafer platform to form a hybrid multifunctional sensor array. Figure 30 shows an example of such a hybrid NO_2_ and CO sensor where the bonding between tungsten oxide (WO_3−x_) NPs and B-UNCD NWs forms the p-n junction. This heterojunction facilitates the electron transfer mobility, given the similar work functions of tungsten oxide (~4.75 eV) and B-UNCD.

When NO_2_ or O_2_ gas molecules adsorb on the surfaces of tungsten oxide NPs, the electrons from the sensor will transfer towards the gas adsorbates and therefore attract more electrons moving from B-UNCD NWs to tungsten oxide, which will increase the NWs’ conductivity. The mobility of electron transport can be enhanced, as well as gas sensitivity. Once the hybrid sensor receives both UV radiation and target gas exposure, UV radiation and gas molecules will take effect on NWs and tungsten oxide NPs, respectively. On the other hand, the NWs will absorb the photonic energy from UV radiation, and the valence electrons will be excited and become conduction electrons. Due to the electron depletion on the NPs’ surface, the electrons from NWs will be pushed towards tungsten oxide NPs through the p-n junction by the diffusion potential. Such induced electron transfer will provide abundant free electrons to the acceptor-gas molecules; meanwhile, it will retain the bandgap for UV photonic absorption.

As indicated by the PZR effect, UNCD NWs demonstrate the electrical resistance change when the NWs are stretched. However, the measured strain is quite small, in the order of 0.01%. Recently, a large and uniform elasticity of the single-crystal diamond NW of 1-mm-long by around 100-nm-wide has been reported, achieving a tensile strain of up to 9.7% [125]. Based on the researchers’ spectroscopy analysis, a reduction of 2 eV in the diamond band structure was observed under such a large elastic strain. Due to the inherent sensing properties and compatibility with nanofabrication processes, this bandgap engineering method might be possibly extended to UNCD NWs for novel applications. On the other hand, although these devices have been demonstrated, physical models and numerical simulations are needed to develop a deep understanding of the mechanisms of these UNCD NW-based devices, and to further optimize the doping effect, the NW geometry and fabrication, the effect of metal nanoparticle surface decoration and nanoplasmonic effect, the electrode design, and the device performance.

## 6. Conclusions

Attributed to the importance of DNWs in diverse semiconductor and biological applications, highly precise UNCD NWs are effectively fabricated using the state-of-the-art wafer fab technologies with a minimum width of 25 nm. In addition, other nanostructures such as vertically aligned nanorods, nanotubes, and nanopillars have also been produced using similar fabrication techniques. The material characterization confirms that the material properties are well preserved after the thin film turns into nanoscale 1D structures. On the other hand, the doping technique effectively reduces the material intrinsic resistance and makes UNCD either a p- or n-type semiconductor. The doped UNCD NWs are catered for developing electrical transduction-based sensors, such as current, potentiometric, resistance/impedance/conductance, while non-conductive UNCD NWs are suited for fluorescence detection. The NW geometry increases the surface-to-volume ratio which enhances sensor performance. In addition, as demonstrated in the UV sensor case, metal nanoparticle surface functionalization is an effective way to further improve the sensor. Although UNCD NW-based gas sensors, UV photodetectors, and PZR sensors have been demonstrated, there is ample room for further improvement and new applications. Additionally, the UNCD NV center-based quantum sensors will open further potential applications with nanoscale resolutions in biological, chemical, and medical fields.

## Figures and Tables

**Table 1:** Property comparison of synthesized SCD and UNCD films. Data from [11,12,13].

Property	SCD	UNCD
Growth Chemistry	H_2_/CH_4_	Ar/CH_4_
Bonding Character	*sp* ^3^	2–5% *sp*^2^
Grain Size (undoped, nm)	1–10,000 (depending on the sample size)	3–5
Grain Size (doped, nm)	-	7–10
Grain Boundary (undoped, nm)	-	~0.4
Grain Boundary (N-doped, nm)	-	1–2
Surface Roughness (nm)	-	4–7
Surface Uniformity (150-mm dia. Si Wafer)		±5%
Density (g/cm^3^)	2.8–3.51	3.30
Poisson Ratio	0.1–0.16	0.057+/−0.038
Young’s Modulus (GPa)	820–900	~850
Hardness (GPa)	100	98
Macroscopic Friction Coefficient in Air	0.01–0.02	0.02–0.05
Dielectric Constant	5.6	5.68
Mohs Hardness	10	10
Intrinsic Resistivity (Ohm-cm)	10^12^–10^16^	10^3^–10^4^

**Table 2:** Comparison of the key device performance of the recently reported thin film and nanowire diamond-based UV PDs. Reprinted with permission from [76]; © 2021, ACS.

Material	Peak λ (nm)	Dark Current	UV/Visible	Responsivity (A/W)	Response Time	Reference
SCD film	220	(NEP ~ 0.5 pW)	10^4^	0.177	-	[86]
SCD film	210	1.1 pA	10^4^	0.048	~80 s	[97]
SCD film	218	5 μA	8.9 × 10^3^	21.8	-	[98]
B-SCD film	210	10 μA	10^6^	230	-	[99]
B-SCD film	220	~1 μA	2 × 10^6^	1	~1 s	[100]
B-SCD film	220	-	10^5^	5.5 × 10^−3^	0.3 s	[81]
B-SCD film	225	1 pA	10^3^	0.028	-	[96]
SCD film	190	(S/N = 10^3^)	10^5^	0.01	160 s	[80]
MCD film	220	5 μA	-	16.2	~20 min	[101]
S-MCD film ^§^ S-SMCD film ^§^ S-NCD film ^§^	220	-	-	0.01	~ms	[83]
PCD film	200	<0.1 nA	10^6^	-	150 ms	[89]
PCD film	220	<0.1 nA	>10^3^	1.625 × 10^−4^	-	[102]
NCD film	365	0.2 mA	-	-	~1 s	[90]
UNCD NW	300	0.07 µA	10^5^	388	20 ms	[76]

^§^ S-: sulfur-doped.

## Data Availability

No new data were created or analyzed in this study. Data sharing is not applicable to this review article.

## Abbreviations

AFM	atomic force microscopy
BOE	buffered oxide etch
B-UNCD	boron-doped ultrananocrystalline diamond
CPD	critical point drying
CVD	chemical vapor deposition
DI	deionized
DNA	deoxyribonucleic acid
DNP	diamond nanoparticle
DNW	diamond nanowire
EBL	electron-beam lithography
EDS	energy-dispersive X-ray spectroscopy
EELS	electron energy loss spectroscopy
FEA	finite element analysis
FESEM	field emission scanning electron microscopy
FE	field emission
FIB	focused ion beam
FWHM	full width at half maximum
HFCVD	hot-filament chemical vapor deposition
HRSEM	high-resolution scanning electron microscopy
HRTEM	high-resolution transmission electron microscopy
HSQ	hydrogen silsesquioxane
ICP	*inductively* coupled plasma
IR	infrared
LER	line edge roughness
MCD	microcrystalline diamond
MIBK	methyl isobutyl ketone
MPCVD	microwave plasma chemical vapor deposition
MSM	metal–semiconductor–metal
NCD	nanocrystalline diamond
ND	nanodiamond
NEMS	nano-electro-mechanical systems
NEXAFS	near-edge X-ray absorption fine structure
NMR	nuclear magnetic resonance
NP	nanoparticle
NR	nanorod
NT	nanotube
N-UNCD	nitrogen-doped ultrananocrystalline diamond
NV	nitrogen-vacancy
NW	nanowire
NWA	nanowire array
PCD	polycrystalline diamond
PD	photodetector
ppm	parts per million
PZR	piezoresistivity/piezoresistance
QIT	quantum information technology
RF	radio frequency
RFCVD	radio frequency chemical vapor deposition
RIE	reactive ion etching
RMS	root-mean-square
RT	room temperature
RTP	a rapid thermal processor
SAED	selected area electron diffraction
sccm	cm³/min in standard conditions for temperature and pressure (a unit of mass flow rate)
SCD	single-crystal diamond
SCL	space-charge-limited
SEM	scanning electron microscopy
SIMS	secondary ion mass spectrometer
S-MCD	sulfur-doped microcrystalline diamond
S-NCD	sulfur-doped nanocrystalline diamond
SOD	spin-on-dopant
S-SMCD	sulfur-doped sub-microcrystalline diamond
STM	scanning tunnel microscopy
TEM	transmission electron microscopy
TLM	transmission line measurement
TMB	trimethyl borane
UHV	ultra-high vacuum
UNCD	ultrananocrystalline diamond
UV	ultraviolet
W	tungsten
WLI	white light interferometer
XRD	X-ray diffraction

## References

[1] Eversole W.G. (1962). Synthesis of Diamond. US Patent.

[2] Spitsyn B.V., Bouilov L.L., Derjaguin B.V. (1981). Vapor growth of diamond on diamond and other surfaces. J. Cryst. Growth.

[3] Butler J.E., Windischmann H. (1998). Developments in CVD-diamond synthesis during the past decade. MRS Bull..

[4] Ong T.P., Chiou W.A., Chen F.R., Chang R.P.H. (1990). Preparation of nanocrystalline diamond films for optical coating applications using a pulsed microwave plasma CVD method. Carbon.

[5] Birrell J., Gerbi J.E., Auciello O., Gibson J.M., Johnson J., Carlisle J.A. (2005). Interpretation of the Raman spectra of ultrananocrystalline diamond. Diam. Relat. Mater..

[6] Gruen D.M. (2001). Ultrananocrystalline diamond in the laboratory and the Cosmos. MRS Bull..

[7] Jiao S., Sumant A.V., Kirk M.A., Gruen D.M., Krauss A.R., Auciello O. (2001). Microstructure of ultranocrystalline diamond films grown by microwave Ar-CH_4_ plasma chemical vapor deposition with or without added H_2_. J. Appl. Phys..

[8] Fuentes-Fernandez E.M.A., Alcantar-Peña J.J., Lee G., Boulom A., Phan H., Smith B., Nguyen T., Sahoo S., Ruiz-Zepeda F., Arellano-Jimenez M.J. (2016). Synthesis and characterization of microcrystalline diamond to ultrananocrystalline diamond films via Hot Filament Chemical Vapor Deposition for scaling to large area applications. Thin Solid Films.

[9] Sumant A.V., Auciello O., Yuan H.-C., Ma Z., Carpick R.W., Maccini D.C. (2009). Large-area low-temperature ultrananocrystalline diamond (UNCD) films and integration with CMOS devices for monolithically integrated diamond MEMS/NEMS-CMOS systems. Proc. SPIE.

[10] Sumant A.V., Auciello O., Carpick R.W., Srinavasan S., Butler J.E. (2010). Ultranocrystalline and nanocrystalline diamond thin films for MEMS/NEMS applications. MRS Bull..

[11] Butler J.E., Sumant A.V. (2008). The CVD of nanodiamond materials. Chem. Vap. Depos..

[12] Advanced Diamond Technologies. http://www.thindiamond.com/wp-content/uploads/2015/06/20120306_Wafers-Data-Sheet.pdf.

[13] Sato Y., Kamo M. (1989). Texture and some properties of vapor-deposited diamond films. Surf. Coat. Technol..

[14] Shellaiah M., Wen Sun K., Rackauskas S. (2019). Diamond Nanowire Synthesis, Properties and Applications. Nanowires-Synthesis, Properties and Applications.

[15] Szunerits S., Coffinier Y., Boukherroub R. (2015). Diamond Nanowires: A Novel Platform for Electrochemistry and Matrix-Free Mass Spectrometry. Sensors.

[16] Yang N., Foord J.S., Jiang X. (2016). Diamond electrochemistry at the nanoscale: A review. Carbon.

[17] Okoli S., Haubner R., Lux B. (1989). Deposition of diamond layers by hot-filament activated CVD using acetone as a carbon source. J. Physique Colloques.

[18] Hirose Y. Synthesis of Diamond using combustion flame in the atmosphere. Proceedings of the 1st International Conference of New Diamond Science and Technology.

[19] Fauchais P., Vardelle A. (1997). Thermal plasmas. IEEE Trans. Plasma Sci..

[20] Davis R.F., Davis R.F. (1993). Diamond Films and Coatings. Development, Properties an Applications.

[21] Liu H., Dandy D.S., William A. (1995). Diamond Chemical Vapor Deposition—Nucleation and Early Growth Stages.

[22] Das D., Singh R.N. (2007). A review of nucleation, growth and low temperature synthesis of diamond thin films. Int. Mater. Rev..

[23] Rotter S. (1996). A novel nucleation method for diamond CVD for conformalcoatings. Diamond Films Technology.

[24] Auciello O., Sumant A.V. (2010). Status review of the science and technology of ultrananocrystalline diamond (UNCD^TM^) films and application to multifunctional devices. Diam. Relat. Mater..

[25] Gruen D.M. (1998). Nucleation, growth, and microstructure of nanocrystalline diamond films. MRS Bull..

[26] Zapol P., Sternberg M., Curtiss L.A., Frauenheim T., Gruen D.M. (2001). Tight-binding molecular-dynamics simulation of impurities in ultrananocrystalline diamond grain boundaries. Phys. Rev. B..

[27] Williams O.A., Zimmermann T., Kubovic M., Denisenko A., Kohn E., Jackman R.B., Gruen D.M., Gruen D.M., Shenderova O.A., Vul A.Y. (2005). Electronic Properties and Applications of Ultrananocrystalline Diamond. Synthesis, Properties and Applications of Ultrananocrystalline Diamond.

[28] Williams O.A., Curat S., Gerbi J.E., Gruen D.M., Jackman R.B. (2004). N-type conductivity in ultrananocrystalline diamond films. Appl. Physics Lett..

[29] Yokoya T., Nakamura T., Matsushita T., Muro T., Takano Y., Nagao M., Takenouchi T., Kawarada H., Oguchi T. (2005). Origin of the metallic properties of heavily boron-doped superconducting diamond. Nature.

[30] Ekimov A., Sidorov V.A., Bauer E.D., Mel’nik N.N., Curro N.J., Thompson J.D., Stishov S.M. (2004). Superconductivity in diamond. Nature.

[31] Tirado P., Alcantar-Peña J.J., de Obaldia E., Kudriavtsev Y., García R., Auciello O. (2018). Boron doping of ultrananocrystalline diamond films by thermal diffusion process. MRS Commun..

[32] Peng X.Y., Chu J., Wang L.D., Duan S., Feng P. (2017). Boron-doped diamond nanowires for CO gas sensing application. Sens. Actuators B Chem..

[33] Liu Y.L., Sun K.W., Lin Y.J., Fong S.-C., Lin I.N., Tai N.H. (2012). Microwave plasma-assisted photoluminescence enhancement in nitrogen-doped ultrananocrystalline diamond film. AIP Adv..

[34] Terranova M.L., Orlanducci S., Rossi M., Tamburri E. (2015). Nanodiamonds for field emission: State of the art. Nanoscale.

[35] Lin C.R., Wei D.H., Bendao M.K. (2014). Effects of Nitrogen Doping on Nanocrystalline Diamond/p-Type Si toward Solar Cell Applications. Adv. Mater. Res..

[36] Yang N., Yu S., Macpherson J.V., Einaga Y., Zhao H., Zhao G., Swain G.M., Jiang X. (2019). Conductive diamond: Synthesis, properties, and electrochemical applications. Chem. Soc. Rev..

[37] Zhang W., Radadia A.D. (2016). Toward a Boron-Doped Ultrananocrystalline Diamond Electrode-Based Dielectrophoretic Preconcentrator. Anal. Chem..

[38] Seo J., Wu H., Mikael S., Mi H., Blanchard J.P., Venkataramanan G., Zhou W., Gong S., Morgan D. (2016). Thermal diffusion boron doping of single-crystal natural diamond. J. Appl. Phys..

[39] Duan H., Winston D., Yang J.K.W., Cord B.M., Manfrinato V.R., Berggren K.K. (2010). Sub-10-nm half-pitch electron-beam lithography by using poly(methyl methacrylate) as a negative resist. J. Vac. Sci. Technol. B.

[40] Cui Z. (2008). Nanofabrication: Principles, Capabilities and Limits.

[41] Advanced Diamond Technologies. http://www.thindiamond.com/wp-content/uploads/2015/06/ADT-Diamond-Etch-Recipe-CMP-Pad.pdf.

[42] Yang N., Uetsuka H., Osawa E., Nebel C.E. (2008). Vertically Aligned Nanowires from Boron-Doped Diamond. Nano Lett..

[43] Babinec T.M., Hausmann B.J.M., Khan M., Zhang Y., Maze J.R., Hemmer P.R., Lončar M. (2010). A diamond nanowire single-photon source. Nat. Nanotechnol..

[44] Sankaran K.J., Haenen K., Yang N. (2019). Nitrogen Incorporated (Ultra)Nanocrystalline Diamond Films for Field Electron Emission Appilcations. Novel Aspects of Diamond: From Growth to Applications (Topics in Applied Physics).

[45] Harniman R.L., Fox O.J.L., Janssen W., Drijkoningen S., Haenen K., May P.W. (2015). Direct observation of electron emission from grain boundaries in CVD diamond by PeakForce-controlled tunnelling atomic force microscopy. Carbon.

[46] Wang X., Ocola L.E., Divan R.S., Sumant A.V. (2012). Nanopatterning of ultrananocrystalline diamond nanowires. Nanotechnology.

[47] Wojick M.J., Joshi V., Sumant A.V., Divan R., Ocola L.E., Lu M., Mancini D.C. (2010). Nanofabrication of x-ray zone plates using ultrananocrystalline diamond molds and electroforming. J. Vac. Sci. Technol. B.

[48] Makarova O., Divan R., Moldovan N., Rosenmann D., Tang C.-M. (2010). Nanoporous ultrananocrystalline diamond membranes. J. Vac. Sci. Technol. B.

[49] Sekaric L., Parpia J.M., Craighead H.G., Feygelson T., Houston B.H., Butler J.E. (2002). Nanomechanical Resonant Structures in Nanocrystalline Diamond. Appl. Phys. Lett..

[50] Chen L.-J., Liu C.-C., Tai N.-H., Lee C.-Y., Fang W., Lin I.-N. (2008). Effects of Tungsten Metal Coatings on Enhancing the Characteristics of Ultrananocrystalline Diamond Films. J. Phys. Chem. C.

[51] Naguib N.N., Elam J.F., Birrell J., Wang J., Grierson D.S., Kabius B., Hiller J.M., Sumant A.V., Carpick R.W., Auciello O. (2006). Enhanced nucleation, smoothness and conformality of ultrananocrystalline diamond (UNCD) ultrathin films via tungsten interlayers. Chem. Phys. Lett..

[52] Wang X. (2012). Synthesis, Fabrication, Characterization and Application of Ultrananocrystalline Diamond Micro- and Nanosctructures. Ph.D. Thesis.

[53] Hausmann B.J.M., Khan M., Zhang Y., Babinec T.M., Martinick K., McCutcheon M., Hemmer P.R., Lončar M. (2010). Fabrication of diamond nanowires for quantum information processing applications. Diam. Relat. Mater..

[54] Mandal S., Naud C., Williams O.A., Bustarret E., Omnes F., Rodiere P., Meunier T., Saminadayar L., Bauerle C. (2010). Nanostructures made from superconducting boron-doped diamond. Nanotechnology.

[55] Zou Y.S., Yang Y., Zhang W.J., Chong Y.M., He B., Bello I., Lee S.T. (2008). Fabrication of diamond nanopillars and their arrays. Appl. Phys. Lett..

[56] Daenen M., Williams O.A., D’Haen J., Haenen K., Nesládek M. (2006). Seeding, growth and characterization of nanocrystalline diamond films on various substrates. Phys. Status Solidi A.

[57] Girard H.A., Perruchas S., Gesset S., Chaigneau S., Vieille L., Arnault J.-C., Bergonzo P., Boilot J.-P., Gacoin T. (2009). Electrostatic Grafting of Diamond Nanoparticles: A Versatile Route to Nanocrystalline Diamond Thin Films. ACS Appl. Mater. Int..

[58] Lee S.-K., Kim J.-H., Jeong M.-G., Song M.-J., Lim D.-S. (2010). Direct deposition of patterned nanocrystalline CVD diamond using an electrostatic self-assembly method with nanodiamond particles. Nanotechnology.

[59] Kromka A., Babchenko O., Rezek B., Ledinsky M., Hruska K., Potmesil J., Vanecek M. (2009). Simplified procedure for patterned growth of nanocrystalline diamond micro-structures. Thin Solid Films.

[60] Chen Y.-C., Tzeng Y., Davray A., Cheng A.-J., Ramadoss R., Park M. (2008). Fabrication of diamond micro-structures by ink-jet printed diamond seeding and microwave plasma assisted chemical vapor deposition. Diam. Relat. Mater..

[61] Hees J., Kriele A., Williams O.A. (2011). Electrostatic self-assembly of diamond nanoparticles. Chem. Phys. Lett..

[62] Yang J.K.W., Berggren K.K. (2007). Using high-contrast salty development of hydrogen silsesquioxane for sub-10-nm half-pitch lithography. J. Vac. Sci. Technol. B Microelectron. Nanometer. Struct..

[63] Gujrati A., Khanal S.R., Pastewka L., Jacobs T.D.B. (2018). Combining TEM, AFM, and Profilometry for Quantitative Topography Characterization across All Scales. ACS Appl. Mater. Int..

[64] Pachiu C., Sandu T., Tibeica C., Avram A., Veca L.M., Popa R., Popescu M., Gavrila R., Popov C., Avramescu V. (2018). Fabrication and characterization of suspended microstructures of ultrananocrystalline diamond. Rom. J. Inf. Sci. Technol..

[65] Chen Y.C., Zhong X.Y., Konicek A.R., Grierson D.S., Tai N.H., Lin I.N., Kabius B., Hiller J.M., Sumant A.V., Carpick R.W. (2008). Synthesis and characterization of smooth ultrananocrystalline diamond films via low pressure bias-enhanced nucleation and growth. Appl. Phys. Lett..

[66] Zhong X.Y., Chen Y.C., Tai N.H., Lin I.N., Hiller J.M., Auciello O. (2008). Effect of pretreatment bias on the nucleation and growth mechanisms of ultrananocrystalline diamond films via bias-enhanced nucleation and growth: An approach to interfacial chemistry analysis via chemical bonding mapping. J. Appl. Phys..

[67] Williams D.F., Marks R.B. (1991). Transmission line capacitance measurement. IEEE Mic. Guid. Wave Lett..

[68] Vlasov I.I., Goovaerts E., Ralchenko V.G., Konov V.I., Khomich A.V., Kanzyuba M.V. (2007). Vibrational properties of nitrogen-doped ultrananocrystalline diamond films grown by microwave plasma CVD. Diam. Relat. Mater..

[69] Panda K., Sundaravel B., Panigrahi B.K., Magudapathy P., Nandagopala Krishna D., Nair K.G.M., Chen H.-C., Lin I.-N. (2011). Structural and electronic properties of nitrogen ion implanted ultrananocrystalline diamond surfaces. J. Appl. Phys..

[70] Birrell J., Carlisle J.A., Auciello O., Gruen D.M., Gibson J.M. (2002). Morphology and electronic structure in nitrogen-doped ultrananocrystalline diamond. Appl. Phys. Lett..

[71] Birrell J., Gerbi J.E., Auciello O., Gibson J.M., Gruen D.M., Carlisle J.A. (2003). Bonding structure in nitrogen doped ultrananocrystalline diamond. J. Appl. Phys..

[72] Gruen D.M., Bruno P., Xie M. (2008). Configurational, electronic entropies and the thermoelectric properties of nanocarbon ensembles. Appl. Phys. Lett..

[73] Feng P., Wang X., Aldalbahi A., Zhou A.F. (2013). Methane induced electrical property change of nitrogen doped ultrananocrystalline diamond nanowires. Appl. Phys. Lett..

[74] Zhou A.F., Wang X., Feng X.P. (2020). Nitrogen-doped Diamond Nanowire Gas Sensor for the Detection of Methane. Adv. Mater. Lett..

[75] Peng X., Li Y., Duan S., Chu J., Feng P. (2020). Precise ultrananocrystalline diamond nanowire arrays for high performance gas sensing application. Mater. Lett..

[76] Zhou A.F., Velázquez R., Wang X., Feng P. (2019). Nanoplasmonic 1D Diamond UV Photodetectors with High Performance. ACS Appl. Mater. Int..

[77] Basu S., Basu P.K. (2009). Nanocrystalline Metal Oxides for Methane Sensors: Role of Noble Metals. J. Sens..

[78] Feng X., Zhang H.X., Peng X.Y., Sajjad M., Chu J. (2011). A novel compact design of calibration equipment for gas and thermal sensors. Rev. Sci. Instrum..

[79] Chu J., Peng X., Sajjad M., Yang B., Feng P.X. (2012). Nanostructures and sensing properties of ZnO prepared using normal and oblique angle deposition techniques. Thin Solid Films.

[80] BenMoussa A., Schühle U., Haenen K., Nesládek M., Koizumi S., Hochedez J.-F. (2004). PIN Diamond Detector Development for LYRA, the Solar VUV Radiometer on Board PROBA II. Phys. Status Solidi (a).

[81] Liao M.Y., Koide Y., Alvarez J. (2006). Photovoltaic Schottky Ultraviolet Detectors Fabricated on Boron-Doped Homoepitaxial Diamond Layer. Appl. Phys. Lett..

[82] Liao M., Koide Y. (2006). High-Performance Metal-Semiconductor-Metal Deep-Ultraviolet Photodetectors Based on Homoepitaxial Diamond Thin Film. Appl. Phys. Lett..

[83] Mendoza F., Makarov V., Weiner B., Morell G. (2015). Solar-Blind Field-Emission Diamond Ultraviolet Detector. Appl. Phys. Lett..

[84] Balducci A., Marinellia M., Milani E., Morgada M.E., Tucciarone A., Verona-Rinati G. (2005). Extreme Ultraviolet Single-Crystal Diamond Detectors by Chemical Vapor Deposition. Appl. Phys. Lett..

[85] Iwakaji Y., Kanasugi M., Maida O., Ito T. (2009). Characterization of Diamond Ultraviolet Detectors Fabricated With High-Quality Singlecrystalline Chemical Vapor Deposition Films. Appl. Phys. Lett..

[86] Teraji T., Yoshizaki S., Wada H., Hamada M., Ito T. (2004). Highly Sensitive UV Photodetectors Fabricated Using High-Quality Single-Crystalline CVD Diamond Films. Diam. Relat. Mater..

[87] Liao M., Wang X., Teraji T., Koizumi S., Koide Y. (2010). Light Intensity Dependence of Photocurrent Gain in Single-Crystal Diamond Detectors. Phys. Rev. B.

[88] Liu K., Liu B., Zhao J., Shu G., Xu X., Yao K., Sun M., Zhang X., Yang Y., Su Z. (2019). Application of back bias to interdigital-electrode structured diamond UV detector showing enhanced responsivity. Sens. Actuators A Phys..

[89] McKeag R.D., Chan S.M., Jackman R.B. (1995). Polycrystalline Diamond Photoconductive Device with High UV-Visible Discrimination. Appl. Phys. Lett..

[90] Lin C.R., Wei D.H., BenDao M.K., Chen W.E., Liu T.Y. (2014). Development of High-Performance UV Detector Using Nanocrystalline Diamond Thin Film. Int. J. Photoenergy.

[91] Zarazua I., Bisquert J., Garcia-Belmonte G. (2016). Light-Induced Space-Charge Accumulation Zone as Photovoltaic Mechanism in Perovskite Solar Cells. J. Phys. Chem. Lett..

[92] Gunkel F., Waser R., Ramadan A.H.H., De Souza R.A., Hoffmann-Eifert S., Dittmann R. (2016). Space Charges and Defect Concentration Profiles at Complex Oxide Interfaces. Phys. Rev. B.

[93] Nesladek M., Stals L.M., Stesmans A. (1998). Dominant Defect Levels in Diamond Thin Films: A Photocurrent and Electron Paramagnetic Resonance Study. Appl. Phys. Lett..

[94] Saguy C., Kalish R., Cytermann C., Teukam Z., Chevallier J., Jomard F., Tromson-Carli A., Butler J.E., Baron C. (2004). N-Type Diamond with High Room Temperature Electrical Conductivity by Deuteration of Boron Doped Diamond Layers. Diam. Relat. Mater..

[95] Liu X., Chen X., Singh D.J., Stern R.A., Wu J., Petitgirard S., Bina C.R., Jacobsen S.D. (2019). Boron-Oxygen Complex Yields N-Type Surface Layer in Semiconducting Diamond. Proc. Natl. Acad. Sci. USA.

[96] Shi X., Yang Z., Yin S., Zeng H. (2016). Al Plasmon-Enhanced Diamond Solar-Blind UV Photodetector by Coupling of Plasmon and Excitons. Mater. Technol..

[97] BenMoussa A., Soltani A., Haenen K., Kroth U., Mortet V., Barkad H.A., Bolsee D., Hermans C., Richter M., De Jaeger J.C. (2008). New Developments on Diamond Photodetector for VUV Solar Observations. Semicond. Sci. Technol..

[98] Lin C.N., Lu Y.J., Yang X., Tian Y.Z., Gao C.J., Sun J.L., Dong L., Zhang F., Hu W.D., Shan C.X. (2018). Diamond-Based All-Carbon Photodetectors for Solar-Blind Imaging. Adv. Opt. Mater..

[99] Alvarez J., Liao M., Koide Y. (2005). Large Deep-Ultraviolet Photocurrent in Metal-Semiconductor-Metal Structures Fabricated on As-Grown Boron-Doped Diamond. Appl. Phys. Lett..

[100] Koide Y., Liao M., Alvarez J. (2006). Thermally stable solar-blind diamond UV photodetector. Diam. Relat. Mater..

[101] Wang L., Chen X., Wu G., Guo W., Wang Y., Cao S., Shang K., Han W. (2010). Study on Trapping Center and Trapping Effect in MSM Ultraviolet Photo-Detector on Microcrystalline Diamond Film. Phys. Status Solidi (a).

[102] Salvatori S., Scotti F., Conte G., Rossi M.C. (1999). Diamond-Based UV Photodetectors for High Temperature Applications. Electr. Lett..

[103] Milne J.S., Rowe A.C.H., Arscott S., Renner C. (2010). Giant Piezoresistance Effects in Silicon Nanowires and Microwires. Phys. Rev. Lett..

[104] Greil J., Lugstein A., Zeiner C., Strasser G., Bertagnolli E. (2012). Tuning the Electro-optical Properties of Germanium Nanowires by Tensile Strain. Nano Lett..

[105] Sumant A.V., Wang X. (2017). Piezoresistive Boron Doped Diamond Nanowire. US Patent.

[106] Middelhoek S., Audet S.A. (1989). Silicon Sensors.

[107] Varney M.W., Aslam D.M., Janoudi A., Chan H.-Y., Wang D.H. (2011). Polycrystalline-Diamond MEMS Biosensors Including Neural Microelectrode-Arrays. Biosensors.

[108] Zhou Y., Zhi J., Zou Y., Zhang W., Lee S.-T. (2008). Direct Electrochemistry and Electrocatalytic Activity of Cytochromec Covalently Immobilized on a Boron-Doped Nanocrystalline Diamond Electrode. Anal. Chem..

[109] Yang W., Auciello O., Butler J.E., Cai W., Carlisle J.A., Gerbi J.E., Gruen D.M., Knickerbocker T., Lasseter T.L., Russell J.N. (2002). DNA-modified nanocrystalline diamond thin-films as stable, biologically active substrates. Nat. Mater..

[110] Vermeeren V., Wenmackers S., Wagner P., Michiels L. (2009). DNA Sensors with Diamond as a Promising Alternative Transducer Material. Sensors.

[111] Matsubara T., Ujie M., Yamamoto T., Akahori M., Einaga Y., Sato T. (2016). Highly sensitive detection of influenza virus by boron-doped diamond electrode terminated with sialic acid-mimic peptide. Proc. Nat. Acad. Sci. USA.

[112] Sankaran K.J., Kunuku S., Lou S.C., Kurian J., Chen H.-C., Lee C.-Y., Tai N.-H., Leou K.-C., Chen C., Lin I.-N. (2012). Microplasma illumination enhancement of vertically aligned conducting ultrananocrystalline diamond nanorods. Nanoscale Res. Lett..

[113] Wang J., Su S., Qiu J., Wang S. (2019). One-Dimensional Fluorescent Nanosized-diamond Nanowires with Fluorescent Detection of Vitamin B_12_. Nano.

[114] Schirhagl R., Kevin C., Loretz M., Degen C.L. (2014). Nitrogen-Vacancy Centers in Diamond: Nanoscale Sensors for Physics and Biology. Ann. Rev. Phys. Chem..

[115] Radtke M., Bernardi E., Slablab A., Nelz R., Neu E. (2019). Nanoscale sensing based on nitrogen vacancy centers in single crystal diamond and nanodiamonds: Achievements and challenges. Nano Futures.

[116] Bukach A.A., Kilin S.Y. (2007). Quantum repeater based on NV + 13C color centers in diamond. Opt. Spectrosc..

[117] Aharonovich I.S., Castelletto S., Simpson D.A., Su C.-H., Greentree A.D., Prawer S. (2011). Diamond-based single-photon emitters. Rep. Prog. Phys..

[118] Su C.-H., Greentree A.D., Hollenberg L.C.L. (2009). High-performance diamond-based single-photon sources for quantum communication. Phys. Rev. A.

[119] Bucher D.B., Glenn D.R., Park H., Lukin M.D., Walsworth R.L. (2020). Hyperpolarization-Enhanced NMR Spectroscopy with Femtomole Sensitivity Using Quantum Defects in Diamond. Phys. Rev. X.

[120] Barry J.F., Schloss J.M., Bauch E., Turner M.J., Hart C.A., Pham L.M., Walsworth R.L. (2020). Sensitivity optimization for NV-diamond magnetometry. Rev. Mod. Phys..

[121] Hatano M., Iwasaki T. Device engineering for diamond quantum sensors. Proceedings of the IEEE International Electron. Devices Meeting (IEDM).

[122] Petkov E., Rendler T., Petkov C., Schnabel F., Reithmaier J.P., Wrachtrup J., Popov C., Kulisch W. (2013). Investigation of NV centers in nano- and ultrananocrystalline diamond pillars. Phys. Status Solid (a).

[123] Stern E., Wagner R., Sigworth F.J., Breaker R., Fahmy T.M., Reed M.A. (2007). Importance of the Debye Screening Length on Nanowire Field Effect Transistor Sensors. Nano Lett..

[124] Gabrysch M., Majdi S., Twitchen D.J., Isberg J. (2011). Electron and Hole Drift Velocity in Chemical Vapor Deposition Diamond. J. Appl. Phys..

[125] Dang C., Chou J.P., Dai B., Chou C.T., Yang Y., Fan R., Lin W., Meng F., Hu A., Zhu J. (2021). Achieving large uniform tensile elasticity in microfabricated diamond. Science.

